# Five‐Year Time Series Reveals Short‐Term Blooms of Planktonic Fungi in a Coastal Mediterranean Site

**DOI:** 10.1111/1758-2229.70154

**Published:** 2025-07-12

**Authors:** Emile Laymand, Pierre E. Galand, François‐Yves Bouget, Lucie Bittner, Fabien Joux

**Affiliations:** ^1^ CNRS, Laboratoire d'Océanographie Microbienne (LOMIC), Observatoire Océanologique de Banyuls Sorbonne Université Banyuls‐Sur‐Mer France; ^2^ Institut de Systématique, Evolution, Biodiversité (ISYEB), Muséum National d'Histoire Naturelle, CNRS, Sorbonne Université, EPHE Université des Antilles Paris France; ^3^ CNRS, Laboratoire d'Ecogéochimie des Environnements Benthiques (LECOB), Observatoire Océanologique de Banyuls Sorbonne Université Banyuls‐Sur‐Mer France; ^4^ Institut Universitaire de France Paris France

**Keywords:** amplicon sequence variants (ASVs), coastal water, diversity, marine Fungi, Mediterranean Sea, temporal survey

## Abstract

Fungi have gained recognition as key organisms within the pelagic marine food webs over the past few decades, with studies showing they constitute a significant proportion of eukaryotes in different marine ecosystems. However, how this proportion varies with time, what triggers fungal blooms, and which fungal clades are involved in those blooms are largely open questions. Here, we used a 5‐year, high‐frequency 18S V4 metabarcoding time series from a well‐documented coastal site of the North West Mediterranean Sea to address these questions. This time series has one of the highest temporal resolutions (up to two samples a week) ever used to investigate marine fungal dynamics. We showed that the dynamics of the fungal relative abundance at this site are mainly chaotic, with short‐term blooms dominated by 41 Amplicon Sequence Variants (ASVs), mainly assigned to Ascomycota. Most of these ASVs are not restricted to the Mediterranean Sea or the marine environment. We found weak links between biotic or abiotic parameters and the relative abundance of Fungi. Our study highlights the relevance of high‐frequency time series to study marine fungal dynamics, as it lowers the risk of aliasing and spurious conclusions.

## Graphical abstract

Fungal dynamics at this northwestern Mediterranean marine site were characterised by short‐term blooms during which fungal ASVs accounted for a high fraction (up to 46%) of eukaryotic reads. The fungal community was dominated by a small number of ASVs, mostly non‐exclusive to the Mediterranean Sea or the marine environment. We found only weak links with biotic and abiotic variables.

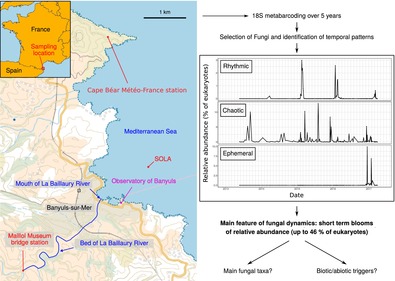

## 1 Introduction

Marine microbial communities strongly drive the global carbon and nutrient cycles in surface waters of the ocean (Falkowski et al. 2008). Since the proposal of the microbial food web concept by Azam et al. (1983), numerous discoveries have highlighted the complexity of the interactions within microbial networks and have revealed an unexpected phylogenetic and functional diversity of their microbial members (Sunagawa et al. 2015; Chen et al. 2024). Understanding the biotic and abiotic mechanisms that control the composition and activities of microbial communities, especially the conditions that trigger or terminate blooms of specific organisms, where high amounts of nutrients and carbon are processed in short amounts of time, is critical to understanding the dynamics of economically‐relevant resources or scourges (e.g., fish stocks, harmful algal blooms), and to correctly estimating biogeochemical mass balances and their variations in an ocean affected by climate change.

Fungi are a component of the pelagic marine community that have long been overlooked. They lack conspicuous morphological features that could be easily identified under the microscope (Peng et al. 2024), but the advent of affordable DNA/RNA sequencing in the late 2000s helped reveal their presence. Fungi can account for a large proportion of the eukaryotic marine plankton (Rojas‐Jimenez et al. 2019; Orsi et al. 2022), and quantitation methods have shown fungal biomass to be important in the upwelling off Chile (Gutiérrez et al. 2011). In the marine realm, four phyla gather most of the fungal sequences throughout the globe: Ascomycota, Chytridiomycota, Basidiomycota, Cryptomycota (Hassett et al. 2020; James et al. 2020). They may act as parasites (e.g., Chytridiomycota on Diatoms) (Gutiérrez et al. 2016) or saprotrophs (e.g., algal polysaccharide degradation by marine‐derived *Cladosporium*) (Cunliffe et al. 2017), although direct evidence of their trophic roles remains limited in the marine environment. Those roles are often extrapolated from their terrestrial or freshwater counterparts (Richards et al. 2012; Taylor and Cunliffe 2016).

Several studies have been conducted with the objective of finding which parameters control the composition of the fungal community in marine pelagic environments (e.g., Duan et al. 2018; Rojas‐Jimenez et al. 2019; Hassett et al. 2020; Chrismas et al. 2023). Several abiotic or biotic parameters (e.g., salinity, dissolved oxygen concentration, phytoplankton biomass) were associated with differences in mycoplankton diversity, yet there is not any agreement on similar parameters (Rojas‐Jimenez et al. 2019; Yang et al. 2021; Sen et al. 2022).

Time series are a valuable tool to infer causality between events occurring at a date (e.g., nutrient inputs from rivers) and responses occurring later (e.g., phytoplankton bloom, then primary consumers bloom) (Romagnan et al. 2015; Martin‐Platero et al. 2018). A few metabarcoding time series have been used to study planktonic Fungi (Duan et al. 2018; Banos et al. 2020; Chrismas et al. 2023). However, due to their Fungi‐specific sequencing protocols, they have mostly been used to explain why fungal communities change in composition over time, but how often and why Fungi represent high proportions of eukaryotes still needs to be investigated.

In this study, we used a 5‐year 18S rDNA metabarcoding time series of sea surface water collected at a coastal site in the NW Mediterranean Sea with sampling frequencies ranging from 2 weeks to 3 days depending on the season. This site is particularly relevant to study sea‐bottom and terrestrial forcings, as it is only 27 m deep, and as it is located 1 km in front of the mouth of an intermittent river that sporadically discharges into the Bay through flood events. This site was previously studied to monitor the temporal evolution of bacterial and phytoplankton communities (Salter et al. 2015; Lambert et al. 2019, 2021). The questions that motivated this study were: (1) Does kingdom Fungi constitute an important proportion of eukaryotes at this site? (2) Which fungal taxa bloom at this site over seasons? (3) What is the duration of these blooms? (4) Which abiotic and biotic parameters trigger these high relative abundance blooms?

## 2 Experimental Procedures

### 2.1 Environmental Sampling and Environmental Parameters

The study took place at the long‐term observatory station SOLA (42°29′366″ N, 3°08′625″ E) in the Bay of Banyuls‐sur‐Mer, northwestern coast of the Gulf of Lions (Mediterranean Sea, France). This 26.3‐m‐deep site is located in the south cove of the Bay, 1 km offshore. The sediment is made of fine sand. The bay is connected to a temporary river, La Baillaury, which flushes abruptly after strong local rainfall.

Surface samples (3 m) were collected with Niskin bottles on board the N/O Nereis II, approximately every 2 weeks from May 2013 to January 2015, twice a week during 2015–2017 winters (January to March 2015, January to April 2016 and December 2016 to March 2017), and once a week during the rest of the years 2015–2017. For each sampling date, the microbial community was collected by filtering a total of 5 L of seawater through two filtering devices in series: a 3‐μm pore‐size polycarbonate filter (Merck‐Millipore, Darmstadt, Germany), then a 0.22‐μm pore‐size GV Sterivex cartridge (Merck‐Millipore). Both filters were stored at −80°C until nucleic acid extraction.

Vertical profiles of salinity and temperature were obtained using a Sea‐bird SBE9/11plus CTD unit (Sea‐Bird Electronics Inc., USA). Nitrate (NO_3_^−^), nitrite (NO_2_^−^), phosphate (PO_4_^3−^) and silicate concentrations were measured using an automated colorimetric technique (Skalar Auto‐Analyser; Tréguer and Le Corre 1975). Chlorophyll *a* concentration was measured using a Turner‐Designs 10‐AU fluorometer (Lorenzen 1967). Phytoplankton cells were analysed by flow cytometry (FacsCalibur, Becton Dickinson).

Unfiltered seawater samples for flow cytometry analysis were fixed at a final concentration of 1% glutaraldehyde, incubated for 15 min at ambient temperature in the dark, frozen in liquid nitrogen and stored at −80°C until analysis. Phytoplankton populations (photosynthetic nano‐ and pico‐eukaryotes, *Synechococcus* and *Prochlorococcus*) were discriminated by their side scatter light diffraction, red fluorescence (measured at 670 nm; chlorophyll content) and orange fluorescence (measured at 585 ± 21 nm, phycoerythrin content) (Salter et al. 2015).

We retrieved the total daily precipitations and the average wind speed at Cape Béar Météo‐France station located ~3.6 km north of SOLA (42°031′ N, 03°008′ E) using Meteo France's online API (https://portail‐api.meteofrance.fr/web/en/api/DonneesPubliquesClimatologie, accessed on 2024‐01‐10). The daily maximal height of La Baillaury River at the Maillol Museum bridge station (station Y010522001, ~3.2 km south‐west of SOLA and ~2.6 km south‐west of the mouth of La Baillaury River) was obtained from the online HydroPortail's interface (https://www.hydro.eaufrance.fr/stationhydro/Y010522001/fiche, accessed on 2024‐02‐20).

### 2.2 DNA Extraction, Amplification and Sequencing

The nucleic acid extraction followed protocols published earlier (Lambert et al. 2019). To summarise, the Sterivex filters were thawed on ice, followed by the addition of lysis buffer (40 nM EDTA, 50 nM Tris, 0.75 M sucrose) and 25 μL of lysozyme (20 mg·mL^−1^).

The filters were then incubated for 45 min at 37°C on a rotary mixer. Subsequently, 8 μL of Proteinase K (20 mg·mL^−1^) and 26 μL of sodium dodecyl sulphate (20% v/v) were added before incubating for 1 h at 55°C. Total DNA was then extracted and purified with the Qiagen AllPrep kit (Qiagen, Hilden, Germany) following the kit's protocol. Specific primers were used to target the eukaryotic V4 region (TAReuk_F1 and TAReuk_R) (Piredda et al. 2017). Library preparation and sequencing were carried out by the Genotoul platform (Toulouse, France), with the Illumina Miseq 2 × 250 bp kits. The raw sequences were deposited in the Sequence Read Archive (SRA) of the NCBI under the accession number PRJNA579489 for the 0.2–3 μm size fraction and under the accession number PRJNA1183754 for the > 3 μm size fraction.

We did not use in the present study the 0.2–3 μm samples for the period 2013–2014 as they had already been sequenced with a different set of primers (Lambert et al. 2019). The comparison of metabarcoding data obtained with different sets of primers is subject to several biases, hence we decided not to consider those samples in the present study.

### 2.3 Sequence Analysis and Preprocessing

The standard pipeline of the DADA2 package (v1.6; Callahan et al. 2016) in ‘R’ was used to do the analysis of the raw sequences. The parameters were: trimLeft = c(20, 21), truncLen = c(250, 250), maxN = 0, maxEE = c(2, 2), truncQ = 2. We analysed 167 and 141 samples for the > 3 μm and the 0.2–3 μm size fractions respectively, and obtained a total of 6.3 and 3.8 million reads respectively after DADA2 data processing. The taxonomic assignment was done with the Protist Ribosomal Reference database (PR2) v.4.10.0 (Guillou et al. 2013) database. The ‘assignTaxonomy’ function in DADA2 implements the RDP naive Bayesian classifier method (Wang et al. 2007). We discarded all samples containing less than 1000 reads (1 sample concerned), leaving for later analysis 166 and 141 samples for the > 3 μm and the 0.2–3 μm size fractions respectively. The samples of the > 3 μm fraction contained on average 37,861 reads (min: 16,914; max: 61,796; sd: 7,482) and on average 1,111 reads were assigned as Fungi (min: 21; max: 20,523; sd: 2,108). The samples of the 0.2–3 μm size fraction contained on average 26,972 reads (min: 1,984; max: 40,864; sd: 6,425) and on average 174 reads were assigned as Fungi (min: 0; max: 4,294; sd: 434).

### 2.4 Data Analysis

Except where mentioned, we analysed the sequencing data using R (v4.3.1). We used the ‘phyloseq’ package (v1.46.0) to conveniently manage and merge data (McMurdie and Holmes 2013).

For each sample, we calculated rarefaction curves using the function ‘rarecurve’ from the package ‘vegan’ (v2.6–4) (Oksanen et al. 2022) to assess the sequencing depth of our samples.

Prior to any further analysis, the raw read counts of each ASV were converted to relative abundance using the following equation:

(1) $${\mathrm{RA}}_{i}^{G,s}=\frac{N_{i}^{s}}{N_{G}^{s}}$$

where ${\mathrm{RA}}_{i}^{G,s}$ is the relative abundance in the sample *s* of taxon *i* normalised by taxon *G* (with taxon *i* contained in taxon *G*), $N_{i}^{s}$ the raw number of sequences assigned to taxon *i* in sample *s*, and $N_{G}^{s}$ the raw number of sequences assigned to taxon *G* in sample *s*. For instance, the relative abundance of ASV *i* in sample *s* is noted ${\mathrm{RA}}_{i}^{\text{Total},s}$ if normalised by the total number of reads in sample *s*, and ${\mathrm{RA}}_{i}^{\text{Fungi},s}$ if normalised by Fungi. Note that ${\mathrm{RA}}_{i}^{\text{Total},s}$ = ${\mathrm{RA}}_{i}^{\text{Eukaryotes},s}$ in all samples, as all reads were assigned to eukaryotes in all samples.

We also defined the Cumulative Relative Abundance (CRA) of a taxon *i* within a taxon *G* (*i* included in *G*) as follows:

(2) $${\mathrm{CRA}}_{i}^{G}=\frac{\sum_{s=1}^{S}{\mathrm{RA}}_{i}^{\text{Total},s}}{\sum_{s=1}^{S}{\mathrm{RA}}_{G}^{\text{Total},s}}=\frac{{\mathrm{CRA}}_{i}^{\text{Total}}}{{\mathrm{CRA}}_{G}^{\text{Total}}}=\frac{\bar{{\mathrm{RA}}_{i}^{\text{Total}}}}{\bar{{\mathrm{RA}}_{G}^{\text{Total}}}}$$

where *S* is the number of samples in the dataset (or in a size fraction, where mentioned) and $\bar{{\mathrm{RA}}_{i}^{G}}$ the average relative abundance across all samples *S* of taxon *i* normalised by taxon *G*. For instance, ${\mathrm{CRA}}_{\mathrm{ASV}224}^{\text{Fungi}}$ is the cumulative relative abundance of ASV224 within Fungi. Concretely, ${\mathrm{CRA}}_{\mathrm{ASV}224}^{\text{Fungi}}$ is the fraction of fungal reads assigned to ASV224, if all samples contained the same number of reads and were merged together. We used this metric as an intuitive tool to evaluate the relevance of fungal sub‐groups (e.g., phyla, ASVs) across the whole dataset: the higher the ${\mathrm{CRA}}_{i}^{\text{Fungi}}$ of a fungal taxon *i*, the more relevant this taxon. A fungal taxon *i* may exhibit a high ${\mathrm{CRA}}_{i}^{\text{Fungi}}$ if it contributes a lot to a couple of peaks of fungal relative abundance, and/or if it consistently represents a higher‐than‐average proportion of Fungi in a large number of samples that exhibit a lower relative abundance of Fungi.

We computed Principal Component Analyses (PCA) using the ‘PCA’ function from the package ‘FactoMineR’ (v2.9) (Lê et al. 2008). As PCA does not handle missing data, we extrapolated missing entries for metadata using the ‘imputePCA’ function from the package ‘missMDA’ (v1.19) (Josse and Husson 2016), with default parameters and ncp = 1. Using this function makes the extrapolated value have no impact on the calculation of the PCA. We centred the mean to 0 and scaled the variance to 1 for each variable prior to PCA computation.

We computed NonMetric Dimensional Scaling (NMDS) to investigate the eukaryotic communities around Fungi by first removing all ASVs assigned to Fungi from the dataset. We then recalculated the relative abundance of each ASV in each sample following Equation (1). We eventually computed the NMDS separately for the > 3 and 0.2–3 μm size fractions using the ‘ordinate’ function from the package ‘phyloseq’ (v1.46.0) (McMurdie and Holmes 2013), with Bray–Curtis dissimilarity used as the metric. The relative abundance values underwent a square root transformation and a Wisconsin double standardisation for the > 3 μm samples, and a Wisconsin double standardisation for the 0.2–3 μm samples prior to the computation of the ordination. The ‘ordinate’ function automatically applied a different transformation for each of the size fractions to improve the final representation, as described in the documentation of the function.

We performed correlations between environmental variables, fungal relative abundances, and the PCA dimensions, and assessed their significance using the function ‘cor_test’ from the package ‘rstatix’ (v0.7.2) (Kassambara 2023). We used the Pearson's r correlation coefficient based on pairwise complete observations and assessed the significance of the correlations with a two‐sided test.

We calculated ${\mathrm{CRA}}_{i}^{\text{Fungi}}$ for each fungal ASV *i* according to Equation (2). More than 90% of ${\mathrm{CRA}}_{\text{Fungi}}^{\text{Total}}$ was represented by 41 ASVs (thereafter called ‘major fungal ASVs’). We curated the taxonomy of these 41 ASVs by manually performing nucleotide BLAST requests against the nt‐nr database via the NCBI's online interface (https://blast.ncbi.nlm.nih.gov/). We kept the lowest taxonomic rank for which all the reference sequences that matched the sequence of the ASV at 100% similarity had the same taxonomy. When we considered BLAST matches to be doubtful (e.g., some Metazoa sequences matching only with Fungi sequences), we performed BLASTs of these dubious sequences onto nt‐nr. We did not take them into account in the curation process if they failed to match sequences from the same taxonomic group (e.g., if a sequence assigned as Metazoa matched only fungal sequences when BLASTed onto nt‐nr).

We investigated the global distribution of the 41 major fungal ASVs using the metaPR2 online interface (https://shiny.metapr2.org/metapr2/, v2.0.1, accessed on 2024‐03‐13) (Vaulot et al. 2022). We considered each of these 41 ASVs as present in a MetaPR2 sample if it matched a sequence from that sample with 100% identity. We used the following parameters for all conditions: V4 region; DNA samples; all size fractions. We used for each environment the following additional combinations of ‘ecosystems’/‘substrates’/‘depth’: Terrestrial: terrestrial/soil/all; Freshwater: freshwater lakes + freshwater rivers/water/all; Coastal: Coastal/water/all; Open Ocean Surface: oceanic/water/surface + euphotic; Open Ocean Deep: oceanic/water/mesopelagic + bathypelagic.

We used ASVs clustered at 100% identity (default parameter) in MetaPR2 to enhance intercomparison between datasets. We required all ASVs to represent at least 100 reads across the whole MetaPR2 database to be considered as present in MetaPR2.

We evaluated the alpha diversity of samples by calculating the Shannon (1948) and Gini‐Simpson (Jost 2007) indices using the function ‘diversity’ from the package ‘vegan’ (v2.6–4) (Oksanen et al. 2022). Unlike what we carried out for most analyses in this study, we normalised here the read counts of fungal ASVs to ${\mathrm{RA}}_{\mathrm{ASV}}^{\text{Fungi}}$ (instead of ${\mathrm{RA}}_{\mathrm{ASV}}^{\text{Total}}$) as the formulae of these alpha diversity indices automatically normalise the read counts of each taxon (here, each fungal ASV) by the sum of all taxa taken into account (hence, the sum of the read counts of all fungal ASVs). We used only samples with at least 50 fungal reads (> 3 μm: 162 samples; 0.2–3 μm: 83 samples). When selecting fungal reads only, the rarefaction curves of most samples with > 50 fungal reads reached a plateau, which indicates that increasing sequencing depth would not modify the results much (Figure S1B,C). We subsequently used the nonparametric Kruskal and Wallis test (Kruskal and Wallis 1952) followed by a post hoc Dunn test (Dunn 1964) with a Benjamini‐Hochberg correction for multiple testing (Benjamini and Hochberg 1995) to examine the significance of the differences of the values of the alpha diversity indices observed between months, seasons and size fractions, with the functions ‘kruskal_test’ and ‘dunn_test’ from the package ‘rstatix’ (v0.7.2) (Kassambara 2023).

To evaluate the periodicity of fungal taxa across the time series, we calculated Lomb‐Scargle periodograms using the ‘lsp’ function from the package ‘lomb’ (v2.1.0) (Ruf 1999), with the following parameters: times = NULL, from = 2, to = 900, type = “period”, ofac = 1, alpha = 0.01, normalise = “standard”.

To identify putative interactions between the 41 major fungal ASVs and the other taxa, we computed a co‐occurrence network using the raw counts of all ASVs of the > 3 μm size fraction using the ‘learn_network’ function of the Julia (v1.5.3) package ‘FlashWeave’ (v0.18.0) (Tackmann et al. 2019), with the following parameters: sensitive = true, heterogeneous = false. FlashWeave automatically normalises taxa counts. We subsequently analysed the network using the R package ‘igraph’ (v2.0.3) (Csárdi et al. 2025). We visually verified the putative interactions using interactive plots produced with the function ‘ggplotly’ from the R package ‘plotly’ (v4.10.4) (Sievert 2020).

To investigate the relationship between the environmental variables and fungal ASVs we computed Canonical Correlation Analyses (CCA) using the ‘cca’ function from the R package ‘vegan’ (v2.6–4) (Oksanen et al. 2022). We used ${\mathrm{RA}}_{\mathrm{ASV}}^{\text{Fungi}}$ of all fungal ASVs as response variables and environmental variables as explanatory variables. We used all samples that contained at least 50 fungal reads. We used all the environmental variables that exhibited a reasonable number of missing values (as CCA do not tolerate them), so that we could use a majority of samples in the computation (137/166 in the > 3 μm size fraction, 66/141 in the 0.2–3 μm size fraction). We centred the mean to 0 and scaled the variance to 1 for each environmental variable prior to computation. We discarded environmental variables from the analysis (NO_3_^−^ in the > 3 μm size fraction, SiO_4_ and NO_3_^−^ in the 0.2–3 μm size fraction) following a Variance Inflation Factors (VIF) analysis using the ‘vif.cca’ function from the R package ‘vegan’ (v2.6–4) (Oksanen et al. 2022). After their removal, all environmental variables exhibited a VIF < 10. We assessed the significance of environmental variables using the ‘anova’ function from the R package ‘vegan’ (v2.6–4) (Oksanen et al. 2022), and considered as significant the *p* values < 0.05.

## 3 Results

### 3.1 The Average Relative Abundance of Fungi Was Low, With Occasional Blooms

**FIGURE 1:**
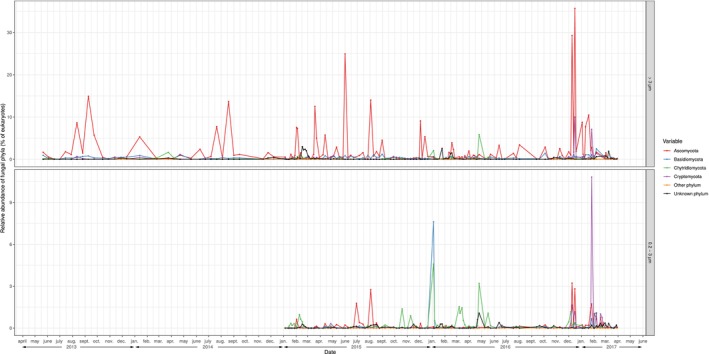
Relative abundance of fungal phyla amongst all eukaryotes across the sampling period. Upper panel: > 3 μm size fraction. Lower panel: 0.2–3 μm size fraction. Colours indicate fungal phyla. Mucoromycota, Entomophtoromycota and Blastocladiomycota are grouped into ‘Other phylum’ and do not represent more than 0.03% of eukaryotes at maximum.

Fungal sequence reads (209,052 reads across all samples, out of 10,088,028 total reads) were clustered into 870 fungal ASVs. All rarefaction curves levelled off, reaching a plateau, indicating that the sequencing depth was sufficient to capture most of the eukaryotic diversity (Figure S1A). Fungal ASVs were taxonomically classified using the PR2 database into 9 ‘classes’, 14 ‘orders’, 27 ‘families’, 170 ‘genera’ and 202 ‘species’. The relative abundances of fungal phyla amongst eukaryotes are displayed in Figure 1.

Overall, ${\mathrm{RA}}_{\text{Fungi}}^{\text{Total}}$ was low, with an average of 1.92% (Figure S2). The average ${\mathrm{RA}}_{\text{Fungi}}^{\text{Total}}$ was lower in the 0.2–3 μm size fraction (0.69%) in comparison to the > 3 μm size fraction (2.96%). The time series exhibited sampling days with high ${\mathrm{RA}}_{\text{Fungi}}^{\text{Total}}$, with a maximum of 46.3% in the larger size fraction, and Fungi was the most abundant domain of eukaryotes in one sample (i.e., 0.3% of samples).

A small number of samples exhibited a high ${\mathrm{RA}}_{\text{Fungi}}^{\text{Total}}$, and represented most of ${\mathrm{CRA}}_{\text{Fungi}}^{\text{Total}}$ (Figure 1). In the > 3 μm size fraction, 17 samples (i.e., 10.2% of the > 3 μm samples) represented more than 50% of ${\mathrm{CRA}}_{\text{Fungi}}^{\text{Total}}$. In the 0.2–3 μm size fraction, 9 samples (i.e., 6.7% of the 0.2–3 μm samples) represented more than 50% of ${\mathrm{CRA}}_{\text{Fungi}}^{\text{Total}}$. Hence, Fungi were present at SOLA station at low relative abundances throughout the year, but they occasionally bloomed to high relative abundances.

${\mathrm{RA}}_{\text{Fungi}}^{\text{Total}}$ was never greater than 10% on two consecutive sampling points, even when the sampling frequency was twice a week. During the periods with 7 days or less between samples, only 4 sampling events with ${\mathrm{RA}}_{\text{Fungi}}^{\text{Total}}$ > 5% were preceded or followed by another point with ${\mathrm{RA}}_{\text{Fungi}}^{\text{Total}}$ > 5% in the > 3 μm size fraction. It was never the case in the 0.2–3 μm size fraction. This highlights the briefness of fungal blooms at this sampling site.

### 3.2 A Small Number of ASVs Dominated the Fungal Community

We assessed the dominance of fungal phyla using ${\mathrm{CRA}}_{\text{Phylum}}^{\text{Fungi}}$. The most dominant fungal phylum overall in both size fractions was Ascomycota (${\mathrm{CRA}}_{\text{Ascomycota}}^{\text{Fungi}}$ = 62.8%), followed by Basidiomycota (12.2%), Chytridiomycota (9.2%), Cryptomycota (8.8%) and Fungi from unknown phylum (7.0%).

In the > 3 μm size fraction, Ascomycota was the most dominant fungal phylum (${\mathrm{CRA}}_{\text{Ascomycota}}^{\text{Fungi}}$ = 70.3%), followed by Basidiomycota (11.6%), Cryptomycota (6.6%), Fungi from unknown phylum (6.4%) and Chytridiomycota (5.1%).

In the 0.2–3 μm size fraction, Chytridiomycota was the most dominant fungal phylum (${\mathrm{CRA}}_{\text{Chytridiomycota}}^{\text{Fungi}}$ = 30.1%), followed by Ascomycota (24.8%), Cryptomycota (20.2%), Basidiomycota (15.2%) and Fungi from unknown phylum (9.8%).

The time‐series fungal community was dominated by a small number of ASVs. Taking into account both size fractions, 5 ASVs represented more than 50% of ${\mathrm{CRA}}_{\text{Fungi}}^{\text{Total}}$, and 41 ASVs (thereafter called ‘major fungal ASVs’) represented more than 90% of ${\mathrm{CRA}}_{\text{Fungi}}^{\text{Total}}$. The taxonomy and ${\mathrm{CRA}}_{\mathrm{ASV}}^{\text{Fungi}}$ of these 41 ASVs as well as complementary information (e.g., sequences of the ASVs, locations where we identified them in MetaPR2) are displayed in Table S1. The dynamics of these 41 ASVs is depicted in Figure S3 (> 3 μm size fraction) and Figure S4 (0.2–3 μm size fraction).

We operationally defined fungal blooms as samples where ${\mathrm{RA}}_{\text{Fungi}}^{\text{Total}}$ ≥ 5%. During fungal blooms, the fungal community was often dominated by a small number of ASVs. Considering both size fractions together, 9.1% of samples were fungal blooms. In 89% of blooms, 2 ASVs or less represented at least 50% of ${\mathrm{RA}}_{\text{Fungi}}^{\text{Total}}$, and no more than 5 ASVs were required to represent at least 50% of ${\mathrm{RA}}_{\text{Fungi}}^{\text{Total}}$. Three ASVs or less represented 90% of ${\mathrm{RA}}_{\text{Fungi}}^{\text{Total}}$ in 43% of blooms, 5 ASVs or less represented 90% of ${\mathrm{RA}}_{\text{Fungi}}^{\text{Total}}$ in 61% of blooms and 32 ASVs or less represented 90% of ${\mathrm{RA}}_{\text{Fungi}}^{\text{Total}}$ in all blooms. Ascomycota were responsible for the majority of blooms considering both size fractions (Figure 1).

### 3.3 The Alpha Diversity of the Fungal Community Was Lower in Summer

We assessed the differences in diversity within the fungal community between seasons and months using the Shannon and Gini‐Simpson indices (Figure S5). Unlike the rest of the analyses, we normalised the read counts of each fungal ASV by the number of fungal reads in the sample (i.e., to ${\mathrm{RA}}_{\mathrm{ASV}}^{\text{Fungi}}$).

We defined winter as January, February and March, as done in a previous publication on the same site (Lambert et al. 2021) and defined other seasons accordingly (e.g., spring: April, May and June). We observed no significant differences between seasons in the 0.2–3 μm size fraction for any of the indices. For the > 3 μm size fraction, we observed significantly lower Gini‐Simpson and Shannon index values in summer compared to autumn and winter.

At the monthly scale, we observed no significant differences between months in the 0.2–3 μm size fraction for any of the indices. In the > 3 μm size fraction, we found that the Gini‐Simpson index was significantly higher in March compared to July, and that the Shannon index was significantly higher in March compared to June, July and August.

We observed significantly higher values for both indices in the > 3 μm size fraction compared to the 0.2–3 μm size fraction.

### 3.4 Rhythmicity and Dynamics of Emergence‐Disparition of Fungi at the ASV Level

We classified the dynamics of the 41 major fungal ASVs into three type categories of rhythmicity depending on their recurrence patterns all along the time series (Figure S6): (1) ‘Rhythmic’ ASVs were ASVs that reappeared at regular frequencies, such as ASV594 that exhibited ${\mathrm{RA}}_{\mathrm{ASV}594}^{\text{Total}}$ peaks every late winter from 2015 to 2017; (2) ‘Chaotic’ ASVs were ASVs that exhibited a high ${\mathrm{RA}}_{\mathrm{ASV}}^{\text{Total}}$ multiple times during the time series, but without any clear temporal pattern, such as ASV224, the fungal ASV with the highest ${\mathrm{CRA}}_{\mathrm{ASV}}^{\text{Fungi}}$; (3) ‘Ephemeral’ ASVs were ASVs that only appeared once in the time series, sometimes on several consecutive sampling days, then never reappeared, such as ASV166 that was only present in noticeable abundances during the 2016–2017 winter. We performed this classification by visually assessing the variations of the relative abundance amongst eukaryotes of these 41 ASVs over the whole time series, in a similar way—albeit not identical—to that used by Banos et al. (2020). We did not aim to make a perfect classification but to evaluate which proportion of these major fungal ASVs exhibited—at least loosely—rhythmic patterns.

Even though some ASVs exhibited clear annual patterns and were termed ‘rhythmic’ by us, we were unable to statistically validate these patterns using Lomb‐Scargle periodograms (data not shown). As a consequence, the rhythmicity of these ASVs must be considered qualitatively, and not as perfectly punctual year after year. These three categories are ideal cases, and many ASVs fell between them (e.g., ASV1991 that was rhythmic on the 2015–2017 period, but not on 2013–2015).

We classified 12 ASVs as—at least partially—rhythmic, with a period of ~1 year. Together, they represented ~13% of ${\mathrm{CRA}}_{\text{Fungi}}^{\text{Total}}$. We assigned 8 of these 12 ASVs to early diverging groups using PR2 (Chytridiomycota, Cryptomycota, unclassified Fungi) and 4 to Ascomycota. A large majority of the major fungal ASVs was non‐rhythmic (~77% of ${\mathrm{CRA}}_{\text{Fungi}}^{\text{Total}}$).

The high sampling frequency during winters from 2015 to 2017 allowed to assess if the fungal ASVs already present on site took time to emerge from low relative abundance populations when favourable conditions appeared in their environment, or if they just appeared and disappeared almost instantly, and were likely imported from elsewhere (e.g., resuspended from sea bottom, imported from land through rivers, imported from farther‐to‐the‐coast waters).

For some ASVs, the peaks were very sharp, their relative abundances being close to 0% on the closest sampling days (3 days before and after). This was the case for ASV275, which formed the first and third highest peaks of fungal relative abundance of the whole time series. On the other hand, some ASVs took up to weeks to build up to their maximum relative abundance and disappear (e.g., ASV594).

### 3.5 Most of the 41 Major Fungal ASVs Were Not Restricted to the Mediterranean Sea nor to the Marine Environment

We searched the MetaPR2 database (Vaulot et al. 2022) to get insights into the biogeography of the 41 major fungal ASVs. More details regarding the sites where we identified these ASVs in MetaPR2 are reported in Table S1.

We found 32 of these 41 ASVs in MetaPR2. We found 27 ASVs (representing 73.9% of ${\mathrm{CRA}}_{\text{Fungi}}^{\text{Total}}$) in other coastal waters, 15 ASVs (52.8% of ${\mathrm{CRA}}_{\text{Fungi}}^{\text{Total}}$) in open ocean surface waters and 21 ASVs (67.5% of ${\mathrm{CRA}}_{\text{Fungi}}^{\text{Total}}$) in open ocean deep waters. We found 12 ASVs (10.6% of ${\mathrm{CRA}}_{\text{Fungi}}^{\text{Total}}$) to be exclusive to the Mediterranean Sea (including 10 exclusive to SOLA). We also found 16 ASVs (53.4% of ${\mathrm{CRA}}_{\text{Fungi}}^{\text{Total}}$) in freshwaters, and 13 ASVs (38.7% of ${\mathrm{CRA}}_{\text{Fungi}}^{\text{Total}}$) in soils. Hence, the distribution area of most of the 41 major fungal ASVs is not restricted to the Mediterranean Sea nor to the marine environment.

We acknowledge that MetaPR2 covers a limited number of locations, although the data compilation effort performed by the database's designers covers most oceanic provinces. As a consequence, the global distribution of these 41 ASVs is likely underestimated, especially for terrestrial and freshwater environments for which the number of locations covered to date by MetaPR2 is limited.

### 3.6 The Dynamics of the Eukaryotic Community Did Not Explain the Variations of the Major Fungal ASVs, nor the Peaks of ${\mathrm{RA}}_{\text{Fungi}}^{\text{Total}}$

We hypothesised that the peaks of ${\mathrm{RA}}_{\text{Total}}^{\text{Fungi}}$ could be associated with a specific assemblage of eukaryotes. To investigate this hypothesis, we computed NMDSs based on all non‐fungal ASVs normalised to ${\mathrm{RA}}_{\mathrm{ASV}}^{\text{Total}\backslash \text{Fungi}}$ (i.e., we normalised their read counts by the number of non‐fungal sequences).

In the > 3 μm size fraction, the eukaryotic communities exhibited an annual and cyclic pattern: samples from the same months and from successive months grouped together year after year (Figure S7A,B). This contrasted with the nonseasonal dynamics of the relative abundance of Fungi.

The 0.2–3 μm size fraction exhibited the same pattern, except for some samples of winter 2016 that clustered with samples of spring (Figure S7C,D). Some samples with the highest relative abundance of Fungi seemed to correspond to outliers (i.e., they were distant from the samples of the same period), namely samples from winter 2015/2016 and 2016/2017 that exhibited a similar composition with samples from spring. Apart from this, apparently a high relative abundance of Fungi as a whole was not associated with a specific microbial community from the same size fraction.

We also investigated the link between the 41 major fungal ASVs and the other eukaryotic ASVs in order to identify potential interactions (e.g., parasitism, saprotrophy on algal material) that could explain the variations of the ${\mathrm{RA}}_{\mathrm{ASV}}^{\text{Total}}$ of these 41 ASVs.

We first focused on ASV224 and ASV1613, that respectively exhibited the 1st and 3rd highest ${\mathrm{CRA}}_{\mathrm{ASV}}^{\text{Fungi}}$ and that represented together nearly one third of ${\mathrm{CRA}}_{\text{Fungi}}^{\text{Total}}$. Both were assigned to Cordycipitaceae (Table S1), a family that contains a large number of arthropod parasites (Pu et al. 2025). As a consequence, we hypothesised that they could be parasites of marine arthropods at SOLA station. To investigate this hypothesis, we visually compared in the > 3 μm size fraction the ${\mathrm{RA}}_{\mathrm{ASV}}^{\text{Total}}$ profiles of these two ASVs and of the ASVs assigned to arthropods that exhibited a ${\mathrm{RA}}_{\mathrm{ASV}}^{\text{Total}}$ > 1% in at least one sample (43 ASVs). However, the profiles did not match well (i.e., peaks of high ${\mathrm{RA}}_{\mathrm{ASV}}^{\text{Total}}$ occurred at distinct moments of the time series).

We expanded our search for potential interactions in a less targeted way by constructing a co‐occurrence network based on the > 3 μm size fraction and considered all edges as putative interactions. All but one of the 41 ASVs (that was not present in the > 3 μm fraction) exhibited at least one direct neighbour (average: 5.5; min: 2; max: 8). These potential interactions were generally weak, with some exceptions (average absolute weight: 0.30; standard deviation: 0.13; max: 1) (Table S2). We visually inspected these potential interactions by plotting together the ${\mathrm{RA}}_{\mathrm{ASV}}^{\text{Total}}$ of the 41 ASVs and their direct neighbours. This inspection revealed that the dynamics of the non‐rhythmic major fungal ASVs and of their direct neighbours only matched weakly (i.e., only some peaks of ${\mathrm{RA}}_{\mathrm{ASV}}^{\text{Total}}$ matched, and very often the fungal ASV exhibited a much higher ${\mathrm{RA}}_{\mathrm{ASV}}^{\text{Total}}$ than its closest neighbours, suggesting that the presence of the fungal ASV was not conditioned by the presence of its neighbours). Notably, the edges with very high weights were spurious and involved ASVs that were present in very few samples. The dynamics of some rhythmic ASVs partially matched the dynamics of some of their neighbours. However, most neighbours exhibited low ${\mathrm{RA}}_{\mathrm{ASV}}^{\text{Total}}$ compared to the fungal ASVs, which implies that the major Fungi likely did not depend on them for growth.

These results show that no single ASV was tightly linked to the peaks of ${\mathrm{RA}}_{\mathrm{ASV}}^{\text{Total}}$ of the 41 major fungal ASVs. This suggests that the 41 major fungal ASVs do not depend on a unique other microbial organism for their growth, and may exhibit opportunistic behaviour.

### 3.7 Abiotic Factors Only Exhibited Weak Links With the Dynamics of Fungi Over the Sampling Period

The dynamics of all environmental parameters are depicted on Figure S8.

August was the month that exhibited the highest proportion of fungal blooms (i.e., samples with ${\mathrm{RA}}_{\text{Fungi}}^{\text{Total}}$ ≥ 5%) (40% of August samples) followed by December (33% of samples) (Figure S2). Conversely, no fungal bloom was observed in May, October and November. These blooms were mainly due to Ascomycota in the > 3 μm size fraction (Figure S9), while phyla contributed more evenly in the 0.2–3 μm size fraction (Figure S10).

We computed Canonical Correlation Analyses (CCA) based on all fungal ASVs normalised to ${\mathrm{RA}}_{\mathrm{ASV}}^{\text{Fungi}}$ to investigate how the variations of environmental variables may explain the changes in the fungal community composition (Figure S11). The environmental variables explained 11% of the inertia in the > 3 μm size fraction, and 22% in the 0.2–3 μm size fraction. The significant driving environmental variables were SiO_4_, chlorophyll a, temperature and NH_4_^+^ in the > 3 μm size fraction, and salinity, NO_2_^−^, chlorophyll a, the height of La Baillaury River and pH in the 0.2–3 μm size fraction.

**FIGURE 2:**
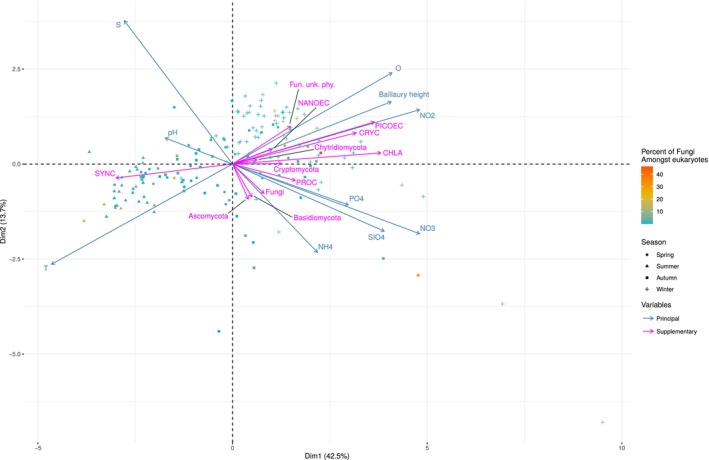
Principal Component Analysis (PCA) based on environmental variables. Blue arrows denote environmental variables that were used to compute the PCA. Purple arrows denote supplementary variables that were projected on the PCA axes but were not used for their calculation. Point colours indicate the percentage of Fungi in the > 3 μm size fraction. Point shapes indicate sampling season. Baillaury height, height of La Baillaury River ~3.2 km south‐west of SOLA; CHLA, chlorophyll *a* concentration; Fun. unk. phy., Fungi from unknown phylum; O, dissolved oxygen; S, salinity; T, temperature; PROC, SYNC, PICOEC, NANOEC and CRYC indicate cell counts for respectively *Prochlorococcus* sp., *Synechococcus* sp., Pico‐eukaryotes, Nano‐eukaryotes and Cryptophyceae. Fungi, Ascomycota, Basidiomycota, Chytridiomycota, Cryptomycota and Fun. unk. phy. refer to the relative abundance of each of these groups amongst eukaryotes.

To investigate if the peaks of ${\mathrm{RA}}_{\text{Fungi}}^{\text{Total}}$ were associated with a specific—or a few specific—combination of environmental parameters, and if the dynamics of ${\mathrm{RA}}_{\text{Fungi}}^{\text{Total}}$ followed the main variations of the environmental variables, we performed a Principal Component Analysis (PCA) based on the environmental variables, and we used biological parameters (relative abundance of fungal ASVs, chlorophyll *a* concentration, flow cytometry counts of phytoplankton) as supplementary variables (Figure 2). The first two PCA axes explained 56.2% of the variance of the environmental variables. Most summer and winter samples clustered respectively together, and constituted two extreme poles with spring and autumn samples located between them. This agrees with the annual pattern observed for the non‐fungal community (Figure S7). Most samples scattered on a line opposing high temperature (i.e., summer) samples to samples obtained in high NO_2_^−^, high dissolved oxygen, high Baillaury height (i.e., winter) conditions. The relative abundance of Fungi as a whole and of fungal phyla projected badly on the main axes of the PCA. Additionally, samples exhibiting a high ${\mathrm{RA}}_{\text{Fungi}}^{\text{Total}}$ did not cluster together. These results suggest that ${\mathrm{RA}}_{\text{Fungi}}^{\text{Total}}$ did not follow the main variations of the environmental parameters and that fungal blooms were not triggered by a unique set of environmental conditions.

After investigating the causes of the variations of the fungal community as a whole, we investigated the links between each of the 41 major fungal ASVs and the environmental variables using correlations (Pearson's r). The 41 major fungal ASVs did not show strong correlations with any of the first 10 principal components of the PCA, nor with any of the environmental variables including chlorophyll *a* and cell counts, although some correlations were significant (Figure S12). This suggests that (1) these ASVs may be under indirect control of the environmental parameters (e.g., under control of other organisms that are themselves under direct control of environmental parameters, see ‘Confounding factors that may hamper the detection of clear patterns of Fungi’), or (2) multiple parameters may control the relative abundance of these ASVs, or (3) parameters that have not been measured may be crucial for these ASVs to bloom.

## 4 Discussion

### 4.1 Using 18S Metabarcodes Is Relevant to Study the Prevalence of Fungi Within Eukaryotes

Unlike what has been done in previous studies relying on time series to investigate Fungi in marine environments (Duan et al. 2018; Banos et al. 2020; Chrismas et al. 2023), we decided to use ${\mathrm{RA}}_{\mathrm{ASV}}^{\text{Total}}$ instead of ${\mathrm{RA}}_{\mathrm{ASV}}^{\text{Fungi}}$ in most of the analyses we carried on. This was made possible by the use of primers that targeted the 18S rRNA gene of all eukaryotes instead of Fungi‐specific primers targeting the ITS region (Duan et al. 2018; Chrismas et al. 2023), likely at the cost of a lower taxonomic resolution at lower taxonomic ranks (e.g., genus and species) (see ‘Accuracy of the taxonomic annotation of Fungal ASVs’), and instead of sequencing procedures involving the inhibition of the amplification of the 18S rRNA genes of non‐fungal groups (Banos et al. 2020).

We think that using the relative abundance of Fungi (or of fungal ASVs) within the whole eukaryotic community gives a more accurate view of their relevance in the ecosystem and their dynamics over time, and is complementary to the information provided by ITS‐based studies.

The normalisation by the total number of reads contextualises better Fungi amongst other eukaryotes. It provides information on their relevance (as a first approximation, the more fungal reads the more important they are) and on the taxa they may interact with (i.e., that are present in the same samples). On the other hand, normalising by the total number of fungal reads only informs on which Fungi exhibit a higher proportion compared to the others; identical proportions may occur regardless of their fraction to the total eukaryotic community, which limits the interpretation of such proportions.

Distinct species are better separated into distinct ASVs using ITS instead of SSU as it is more variable (Heeger et al. 2018). However, because of this same reason, the taxonomic assignment of ITS‐based ASVs is heavily dependent on the presence of close relatives in the reference database, whose absence results in the ASVs being tagged as ‘unknown’ even at the highest taxonomic ranks (i.e., phylum and even kingdom) (Heeger et al. 2018, 2019). Using long reads where both markers were present, Heeger et al. (2018) showed that in their freshwater and sediment samples, 75% of the reads assigned as Fungi using SSU were not assigned at the kingdom level using ITS. This absence of assignment is particularly important for basal lineages (e.g., Chytridiomycota, Chytridiomycota) (Heeger et al. 2018), which are underrepresented in reference databases. This suggests that SSU is the current best short‐reads marker to assign a maximum of fungal reads at higher taxonomic ranks.

Normalising read counts by the total number of reads in each sample presents the major advantage of being independent from any taxonomic assignment. Normalising by the number of fungal reads assumes that all fungal reads have been correctly assigned at the kingdom level. If any major fungal group is unassigned, all normalised counts—and the conclusions drawn out of them—will be skewed. One could argue that the use of Fungi‐specific primers solves this issue, but recent evidence supports that such primers also amplify metazoans and protists (Chrismas et al. 2023). Given that basal phyla have been shown to represent a high fraction of fungal reads in several marine studies (Rojas‐Jimenez et al. 2019; Priest et al. 2021), we consider that using SSU metabarcodes normalised by the total number of reads is a more reliable method to investigate the dynamics of Fungi over time and the causes of these variations compared to using ITS (or Fungi‐specific) metabarcodes normalised by the number of fungal reads.

ITS however remains the best choice to access the lowest levels of taxonomy. Its enhanced taxonomic resolution permits more accurate hypotheses regarding the roles of fungal taxa in the environment, as members of a species likely play less diverse roles compared to members of a whole family. The better discriminating power of ITS compared to 18S also allows for a better description of the alpha diversity of the fungal community. Long reads metabarcoding, that targets the whole rRNA operon (as performed by Heeger et al. 2018), may be an efficient solution to couple the advantages of SSU and ITS metabarcoding.

### 4.2 Conclusions Drawn From Metabarcoding Data Must Be Interpreted With Caution due to the Variable Number of Copies of the rRNA Operon Per Cell

One may argue that the number of copies of the rRNA operon varies too much between eukaryotic kingdoms to use 18S read counts normalised by the total number of reads in a sample as a reliable proxy of the actual proportion of Fungi, and that the use of Fungi‐specific primers with a normalisation by the number of fungal reads at least gives an accurate view of the composition of the fungal community (assuming all fungal reads are correctly assigned at the kingdom level, as mentioned above). Recent evidence supports that the number of copies of the rRNA operon varies over two orders of magnitude within kingdom Fungi (10^2^–10^4^ copies·cell^−1^) (Lofgren et al. 2019). This range is the same in animals and plants and is similar to that observed in diatoms (10^3^–10^4^ copies·cell^−1^), but is lower than that observed in ciliates (10^3^–10^5^ copies·cell^−1^), foraminifera (10^2^–10^5^ copies·cell^−1^) and especially dinoflagellates (e.g., 10^2^–10^8^ copies·cell^−1^ in the genus *Alexandrium*) (Ruvindy et al. 2023). As a consequence, both normalisation methods likely cause skewed results.

This distortion of the actual biomass of organisms may be mitigated by the correlation between the number of rRNA operon copies and the cell volume for some—but not all—marine taxa (Ruvindy et al. 2023). Many conclusions regarding the environmental factors affecting fungal assemblages were drawn from metabarcoding studies (as reviewed by Sen et al. (2022)) and are therefore subject to this number of copies bias. As a consequence, we think that such conclusions must be interpreted with caution. This caveat also applies to the present study.

### 4.3 High ${\mathrm{RA}}_{\text{Fungi}}^{\text{Total}}$ Values at SOLA Are Consistent With an Earlier 18S‐Based Study

Using primers targeting the 18S rRNA gene, Rojas‐Jimenez et al. (2019) observed that the percentage of eukaryotic sequences belonging to kingdom Fungi was on average 5.91% (range 0.23–30.5) and 7.77% (range 0.26–68.1) in two datasets respectively sampled in the Baltic Sea and the Eastern North Sea. As in our study, they often recovered a high percentage of Fungi in their samples, but they showed that Chytridiomycota and Cryptomycota dominated most of these Fungi‐rich samples. This strongly contrasts with our results, as Ascomycota dominated the fungal community in our Fungi‐rich samples. This difference might be due to the characteristics of the sampling locations, but also to technical reasons such as the amplified rDNA region (V7 and V8 in Rojas‐Jimenez et al. (2019), V4 here), the reference database used in the taxonomic assignment (SILVA SSU Ref dataset in Rojas‐Jimenez et al. (2019), PR2 here) and/or the annotation pipeline used. These processing disparities are an obstacle to comparing studies, and hamper understanding the true relationship between environmental conditions and fungal relative abundance. We advocate for complementary studies that would use several primer pairs to compare the differences in observed diversity depending on the marker, to improve comparability between existing studies.

Here, we showed that more than 90% of ${\mathrm{CRA}}_{\text{Fungi}}^{\text{Total}}$ was represented by only 41 ASVs. An earlier study has demonstrated that Fungi (Ascomycota and Basidiodmycota) were underrepresented in metabarcoding samples using the V4 region of the 18S rDNA as a marker gene compared to other regions (e.g., V9) (Obiol et al. 2020). Hence, the fungal relative abundance amongst eukaryotes at SOLA station, yet already high on several dates, might have been underestimated. This is encouraging and calls for additional studies at SOLA station, where the role of Fungi may be even greater than suggested by the data presented here.

### 4.4 Accuracy of the Taxonomic Annotation of Fungal ASVs

We double‐checked the PR2 taxonomy of the 41 major fungal ASVs by blasting the sequences on the nt‐nr database of the NCBI. In many cases for Ascomycota, we obtained exact matches (100% sequence similarity, 100% query cover) on several species. While we considered the taxonomic assignment of some nr‐nt sequences as dubious (e.g., these sequences exhibited low score alignment with other sequences from the clade they were supposed to belong to), many ASVs exhibited exact matches with sequences obtained from type material, which taxonomy is harder to challenge. We often ended up not being able to be more accurate than the family or the sub‐family level. This likely limits the interpretation we can draw from our results regarding the potential trophic roles of the ASVs we retrieved, as a single ASV may actually group multiple organisms with different enzymatic capacities or host‐specific pathogenicities.

For ‘basal’ groups (namely Chytridiomycota and Cryptomycota), our manual taxonomic curation using BLAST showed that they could hardly be identified to a taxonomic level better than phylum or order with the set of primers we used, and most sequences that matched with them at high sequence similarity values were uncultured organisms from metabarcoding surveys. Chytridiomycota and Cryptomycota were shown to be abundant members of the marine pelagic fungal community in a number of environments (Richards et al. 2015, Rojas‐Jimenez et al. 2019; Hassett et al. 2020). As a consequence, there is a real need to fill 18S databases with curated sequences from well identified organisms belonging to these phyla in order to improve the taxonomic resolution of metabarcoding on these groups, that is necessary to infer a reliable functional role for them.

### 4.5 Links Between the Environmental Variables and the Composition of the Fungal Community

Two marine time series located at Beaufort, North Carolina, USA (Duan et al. 2018), and Plymouth, Devon, United Kingdom (Chrismas et al. 2023) exhibited a higher alpha diversity of the fungal community in winter compared to the other seasons. These results are consistent with what we observed at SOLA in the > 3 μm size fraction. A higher alpha diversity in winter within the fungal community hence appears to be a consistent pattern in temperate surface pelagic waters.

Several studies have suggested that multiple environmental parameters may shape the fungal diversity of the water column, the most often reported parameters being phytoplankton, primary production, nutrients, salinity, organic matter, seasonality, dissolved oxygen and temperature (Sen et al. 2022). This is consistent with the results of our CCA where temperature, salinity, nutrients, chlorophyll a, and the height of La Baillaury River were significant drivers of the variations of the fungal community composition, yet the environmental variables only explained between 11% and 22% of the variation observed in the fungal community at SOLA. These values are comparable to the fraction of archaeal, bacterial, and eukaryotic phytoplanktonic variability explained by environmental variables at the same site (7%–14%) (Lambert et al. 2019). Chrismas et al. (2023) reported that the environmental variables only explained ~3% of the variations observed in the fungal community over their 17‐year metabarcoding time series. Environmental variables hence seem to consistently exhibit a weak relationship—that may suggest an indirect control—on the composition of the fungal community over time. This contrasts with the results obtained by Rojas‐Jimenez et al. (2019) in a spatial survey carried out in the Baltic Sea, where they observed clearly distinct community compositions depending on salinity. This suggests that much more important gradients (~30 PSU in Rojas‐Jimenez et al. (2019) vs. ~4 PSU here) may be required to observe a strong relationship between environmental variables and the composition of the fungal community.

### 4.6 Confounding Factors That May Hamper the Detection of Clear Patterns of Fungi

The relative abundance of many of the most prevalent fungal ASVs (e.g., ASV224, ASV1016, ASV1613) exhibited irregular patterns that did not show clear links with environmental parameters. Only 12 ASVs were rhythmic (without statistical support) and together represented ~13% of ${\mathrm{CRA}}_{\text{Fungi}}^{\text{Total}}$. This contrasts with phototrophic pico‐eukaryotes, bacteria and archaea, for which a small number of ASVs (1%, 3.1% and 3.4% respectively) were highly rhythmic (1‐year period, with statistical support) but represented a high fraction of total reads (31.3%, 31.6% and 75.5%, respectively) at SOLA station (Lambert et al. 2019). In addition to these highly rhythmic ASVs, many more may exhibit looser rhythmicity (like the fungal ASVs we labelled as rhythmic). Hence the proportion of rhythmic phototrophic pico‐eukaryotes, bacteria and archaea may be even greater, in strong contrast with Fungi. The irregular fungal patterns could emerge from multiple causes:

1 Saprophytic Fungi often possess large enzymatic arsenals (Girard et al. 2013; Bahram and Netherway 2022). As a consequence, a single fungal species can often use a wide variety of substrates as a food source. For instance, in the marine environment, a single fungal species may feed on phytoplankton but also on metazoan debris, or on organic carbon from sediments or from riverine inputs. As a consequence, multiple causes could trigger blooms of this species, which would prevent the existence of a clear correlation between the relative abundance of this species and a single environmental parameter. Such a signal would be even less clear if two or more parameters were required to change concomitantly for the Fungi to bloom (e.g., an increase of a food source and a source of a vital vitamin). In contrast, the more rhythmic heterotrophic bacteria and archaea may be more tightly linked to a single food source (e.g., rhythmic photoautotrophs) (Lambert et al. 2019).
2 Micro‐heterotrophs do not necessarily instantly react to their food source (e.g., phytoplankton), but may lag behind it for a period of time (Romagnan et al. 2015). As a consequence, we think that the key to explain the dynamics of Fungi may reside in taking into account the local ecological successions and the food webs (Romagnan et al. 2015). For instance, an input of nutrients may cause a bloom of dinoflagellates, that would in turn cause copepods to bloom, and eventually would induce fungal parasites of copepods to emerge. Taking into account successions requires a clearer taxonomic resolution than the one of the 18S V4 primers we used, as clear biotic interactions may be confounded by multiple genera being grouped under the same ASV (see section ‘*Accuracy of the taxonomic annotation of Fungal ASVs*’). Some studies used ITS primers that enhanced the taxonomic resolution they could obtain (up to genus level) (Chrismas et al. 2023); however, we advocate for primers that amplify the whole eukaryotic community instead of Fungi‐specific primers, as we think interpreting fungal blooms within the context of the whole microbial community helps reveal their major roles within the ecosystem (see section ‘Using 18S metabarcodes is relevant to study the prevalence of Fungi within eukaryotes’). One could also think about coupling ITS metabarcoding, Fungi‐specific 18S qPCR and whole‐eukaryotes 18S qPCR to estimate the proportion of Fungi within the Eukaryotes without losing the taxonomic precision of ITS primers. Considering ecological successions would also demand a clear knowledge of the actual ecology of the organisms involved from the data analyst to avoid investigating thousands of putative interactions for every single actual one. Unsupervised machine learning techniques may help to grasp hard‐to‐identify patterns or combinations of parameters that cause Fungi to soar (Hernández Medina et al. 2022), that may be missed by correlation‐based techniques including co‐occurrence networks (Carr et al. 2019). Integrating these upgrades into long term or high‐frequency time series may not be a straightforward task. Fungi are often not the main target of time series—as it is the case for our study—because they usually are not the main components of marine microbial communities.
3 Fungi are often not the primary components of the eukaryotic community. The compositional nature of metabarcoding (Gloor et al. 2017) prevents us from concluding if the fungal blooms we observed were due to real increases in fungal biomass or to the collapse of other groups. Conversely, we cannot know if during periods with lower fungal relative abundance the fungal biomass was actually lower, or if it was similar or even higher but increased less than other groups. This information is however crucial as Fungi may play an important role even if it is not the most dominant group. qPCR and/or fungal biomass quantification may help leverage this issue, albeit they also come with their own technical limitations (e.g., only some groups targeted, reliability of the extrapolations from cell counts to biomass) (Hassett et al. 2019; Priest et al. 2021).

### 4.7 Plausibility That the Major Fungal ASVs Are Active at SOLA Station

Our data evidenced that fungal ASVs often formed peaks of ${\mathrm{RA}}_{\mathrm{ASV}}^{\text{Total}}$ shorter than 3 days. We investigated the possibility that these ASVs reached high relative abundances without external inputs of genomic material (i.e., the peak observed was only due to the multiplication of individuals that were already present in SOLA surface waters, and not to individuals imported from land, sea bottom sediment or offshore waters).

Making the strong assumption that the only organism whose real abundance is changing between time 0 and time *t* is the one represented by the ASV_i_, the specific growth rate this organism must exhibit to experience a multiplication by *x* of its relative abundance in *t* hours can be written as Equation (3):

(3) $$\mu =\frac{1}{t}\log_{2}\left(\frac{x+1}{1-\mathrm{xy}}-1\right)$$

with *μ* the specific growth rate in h^−1^ of the organism represented by the ASV_*i*_, *t* the time spent in hours, and *y* the proportion of eukaryotic cells belonging to the organism represented by the ASV_*i*_ at time 0.

One of the biggest ${\mathrm{RA}}_{\mathrm{ASV}}^{\text{Total}}$ increases we observed for a fungal ASV was the shift of ASV275 from 0% (i.e., the sequencing depth was not high enough to detect any read for this ASV) to 25% between 2016‐12‐13 and 2016‐12‐16. The detection limit on 2016‐12‐13 was 2.5·10^−3^% of eukaryotes (1 read in 39,035). Assuming an initial number of cells belonging to ASV275 10 times lower than detection limit (i.e., *y* = 2.5·10^−6^), this implies *x* = 10^5^. Then, for such a cell multiplication in 3 days, it requires *μ* = 0.23 h^−1^. This would imply that those Fungi exhibit sharp r‐strategies.

Tamminen et al. (2020) showed that the specific growth rate of eight species of marine Fungi cultivated at 24°C and 28 PSU spanned from 0.01 to 0.27 h^−1^ depending on the organic carbon substrate provided. Priest et al. (2021) reported a growth rate of 0.045 h^−1^ for basidiomycetous yeasts during a spring phytoplankton bloom in the North Sea, the highest in situ fungal growth rate reported to date to our knowledge. As a consequence, the values we obtained here in the most extreme cases are theoretically possible, but in the higher range of the abovementioned studies.

The assumptions we made are strong (only one group varying with no loss for this group, no environmental limitations), and may not hold in real in situ conditions. Our calculation nonetheless shows that one cannot rule out that already‐present‐on‐site Fungi may bloom in very short amounts of time, and that peaks of fungal relative abundance may not only be due to fungal cells being imported from land, water bottom or high‐sea waters. It also shows that one must perform sampling at very high frequency (e.g., every day, as performed by Priest et al. 2021) to be able to catch the dynamics of Fungi in coastal areas, which may prove unfeasible on such long‐term time series. Lower frequency time series may exhibit aliasing and may lead to erroneous conclusions (e.g., considering that Fungi are irrelevant at a given sampling site if no fungal bloom is sampled, which would be the most frequent case according to our data, or considering that Fungi may be abundant organisms over long periods of time if several blooms are sampled by chance). Taylor and Cunliffe (2016) already observed successions of bloom‐forming mycoplankton orders at a monthly scale in a coastal station off Plymouth (UK). However, one cannot guarantee that a higher sampling frequency would not have resulted in different conclusions regarding the dynamics of these orders.

Although these abundant ASVs may already be present in the water column at SOLA and bloom when the environmental conditions are favourable, one cannot rule out either that they may be imported from land (only ~1 km away) or from the sediment (only 27 m below the sampling point). Additionally, our calculation showed that the specific growth rates required for some blooms to occur are multiple fold higher than any in situ value ever reported for Fungi in marine environments (Priest et al. 2021). This suggests that at least some blooms may be due to important inputs of allochthonous fungal cells. The water at SOLA station is not still, and undergoes wind‐driven currents (Grémare et al. 2003; Guizien et al. 2006). A plausible hypothesis is that those currents import Fungi from the sediment to surface waters (causing fungal blooms) and subsequently carry them rapidly towards the open sea (causing the abrupt end of the fungal blooms). This hypothesis is consistent with the briefness of the fungal blooms and the weak links we found with both biotic and abiotic variables. This hypothesis is also supported by the strong peaks *of* ${\mathrm{RA}}_{\text{Fungi}}^{\text{Total}}$ during winter 2016–2017 (Figure 1) and the associated high turbidity (Figure S8). However, the records of turbidity at SOLA are irregular and only cover a short timescale; hence, they do not allow to confirm this hypothesis. Under this hypothesis, ASV224 and ASV1613 may be parasites of benthic arthropods, hence the weak links with arthropods we found in our planktonic data.

We retrieved most of the major fungal ASVs from non‐coastal sites in MetaPR2, which suggests an important connectivity between environments. This is not surprising as many terrestrial and freshwater species have been frequently isolated from marine environments (Overy et al. 2014; Jones et al. 2019). As the coverage of terrestrial and freshwater environments in MetaPR2 is low, the already‐high number of major fungal ASVs we identified as not restricted to the Mediterranean Sea nor to the marine environment is probably well underestimated. This connectivity further supports that some blooms may be due to inputs of allochthonous fungal cells. As a consequence, additional sampling must be performed on land near the Bay of Banyuls and in the bottom sediment to confirm or infirm that these abundant fungal ASVs can also be found there, and eventually conclude on the land‐sea and sediment‐pelagos connectivity of the fungal communities at SOLA.

Regardless of the origin of these fungal ASVs, metatranscriptomes must be produced to confirm these ASVs are actually active at SOLA and which metabolic pathways they rely on at this site.

## 5 Conclusion

Our study of a 5‐year high frequency, 18S rDNA metabarcoding time series at SOLA station revealed short‐term blooms of fungal relative abundance that were often dominated by a few ASVs. Most of these ASVs did not exhibit clear seasonal patterns, and we observed weak relationships between their relative abundance and the abiotic and biotic variables. Our study highlights the relevance of high frequency sampling for metabarcoding time series, as lower sampling frequencies might fail to capture the sharp fluctuations of fungal relative abundance. Metatranscriptomics are required to verify that the dominant fungal groups identified in this study are metabolically active, and to further explore their ecological roles within the coastal planktonic microbial community. Extensive sampling of the bottom sediment and of the surrounding terrestrial areas is also necessary to establish if the dominant fungal groups are autochthonous to surface waters or allochthonous.

## Author Contributions

**Emile Laymand:** investigation, writing – original draft, writing – review and editing, conceptualization, methodology, software, funding acquisition. **Pierre E. Galand:** writing – review and editing, conceptualization, funding acquisition, software, resources, data curation. **François‐Yves Bouget:** conceptualization, writing – review and editing, funding acquisition, resources, data curation. **Lucie Bittner:** writing – original draft, writing – review and editing, methodology, investigation, supervision, funding acquisition. **Fabien Joux:** writing – original draft, writing – review and editing, methodology, investigation, supervision, funding acquisition.

## Conflicts of Interest

The authors declare no conflicts of interest.

## Supporting information


**Figure S1.** Rarefaction curves (A) All reads—not only the reads classified as Fungi—are considered. All samples are displayed. (B) Fungal reads only. Only samples with more than 50 fungal reads are displayed. (C) Fungal reads only. Only samples that contain between 50 and 200 fungal reads are displayed. Left panels: > 3 μm size fraction. Right panels: 0.2–3 μm size fraction.

**Figure S2.** Percent of Fungi amongst eukaryotes in function of the month of sampling. (A) > 3 μm size fraction. (B) 0.2–3 μm size fraction. Please note the difference of the *y*‐axis scales of panels (A) and (B). The upper and lower limits of the boxes correspond to the first and third quartiles. The horizontal line in the boxes is the median. The upper (respectively lower) whisker extends from the upper (resp. lower) limit of the box to the largest (resp. smallest) value no further than 1.5 times the inter‐quartile range from the upper (resp. lower) limit of the box. Points with values beyond the end of the whiskers are outliers and are plotted as circles.

**Figure S3.** Dynamics of the 40 major fungal ASVs present in the > 3 μm size fraction. For each ASV, the value displayed in the ‘Max’ column (purple scale) indicates the maximal ${\mathrm{RA}}_{\mathrm{ASV}}^{\text{Total}}$ (noted max(${\mathrm{RA}}_{\mathrm{ASV}}^{\text{Total}}$), in % of total reads) this ASV exhibited in this size fraction. For each ASV, we normalised every ${\mathrm{RA}}_{\mathrm{ASV}}^{\text{Total}}$ value in the time series by max(${\mathrm{RA}}_{\mathrm{ASV}}^{\text{Total}}$) in order to use the same colour scale for all ASVs. The resulting value ${\mathrm{RA}}_{\mathrm{ASV}}^{\text{Total}}$/max(${\mathrm{RA}}_{\mathrm{ASV}}^{\text{Total}}$) is displayed with a red scale.

**Figure S4.** Dynamics of the 36 major fungal ASVs present in the 0.2–3 μm size fraction. For each ASV, the value displayed in the ‘Max’ column (purple scale) indicates the maximal ${\mathrm{RA}}_{\mathrm{ASV}}^{\text{Total}}$ (noted max(${\mathrm{RA}}_{\mathrm{ASV}}^{\text{Total}}$), in % of total reads) this ASV exhibited in this size fraction. For each ASV, we normalised every ${\mathrm{RA}}_{\mathrm{ASV}}^{\text{Total}}$ value in the time series by max(${\mathrm{RA}}_{\mathrm{ASV}}^{\text{Total}}$) in order to use the same colour scale for all ASVs. The resulting value ${\mathrm{RA}}_{\mathrm{ASV}}^{\text{Total}}$/max(${\mathrm{RA}}_{\mathrm{ASV}}^{\text{Total}}$) is displayed with a red scale.

**Figure S5.** Alpha diversity within kingdom Fungi in function of the month of sampling. Indices used are the Gini‐Simpson index (1 − D) in (A) the > 3 μm and (B) the 0.2–3 μm size fractions, and the Shannon index (H′) in (C) the > 3 μm and (D) the 0.2–3 μm size fractions. Colours indicate the season of sampling. Only samples with at least 50 fungal reads were considered in calculations.

**Figure S6.** Relative abundance amongst eukaryotes in the > 3 μm size fraction of (A) ASV594 (‘rhythmic’), (B) ASV224 (‘chaotic’) and (C) ASV166 (‘ephemeral’).

**Figure S7.** NMDS computed with non‐fungal ASVs only, using Bray–Curtis dissimilarity directly at the ASV level. (A) and (C) Colours indicate the month in which the sample was collected, respectively for the > 3 μm size fraction and the 0.2–3 μm size fraction. (B) and (D) Colours indicate the percent of Fungi amongst eukaryotes in the sample, respectively for the > 3 μm size fraction and the 0.2–3 μm size fraction.

**Figure S8.** Variations over the sampling period of the environmental parameters. (A) Temperature, (B) Salinity, (C) Dissolved oxygen, (D) pH, (E) NH_4_^+^ concentration, (F) NO_3_^−^ concentration, (G) NO_2_^−^ concentration, (H) PO_4_^3−^ concentration, (I) (SiO_4_)^4−^ concentration, (J) The height of La Baillaury river ~3.2 km south‐west of SOLA, (K) Daily rainfall at Cape Béar, (L) Average daily wind speed at 10 m at Cape Béar, (M) Turbidity at 3 m below surface, (N) Turbidity at 20 m below surface (~6 m above seafloor), (O) Chlorophyll *a* concentration, (P) *Synechococcus* sp. cell count, (Q) *Prochlorococcus* sp. cell count, (R) Pico‐eukaryotes cell count, (S) Nano‐eukaryotes cell count, (T) Cryptophyceae cell count.

**Figure S9.** Relative abundance amongst Eukaryotes of (A) Ascomycota, (B) Basidiomycota, (B) Chytridiomycota, (D) Cryptomycota and (E) Fungi from unknown phylum, per month over the 5 years of sampling in the > 3 μm size fraction. The upper and lower limits of the boxes correspond to the first and third quartiles. The horizontal line in the boxes is the median. The upper (respectively lower) whisker extends from the upper (resp. lower) limit of the box to the largest (resp. smallest) value no further than 1.5 times the inter‐quartile range from the upper (resp. lower) limit of the box. Points with values beyond the end of the whiskers are outliers and are plotted as circles.

**Figure S10.** Relative abundance amongst Eukaryotes of (A) Ascomycota, (B) Basidiomycota, (C) Chytridiomycota, (D) Cryptomycota and (E) Fungi from unknown phylum, per month over the 5 years of sampling in the 0.2–3 μm size fraction. The upper and lower limits of the boxes correspond to the first and third quartiles. The horizontal line in the boxes is the median. The upper (respectively lower) whisker extends from the upper (resp. lower) limit of the box to the largest (resp. smallest) value no further than 1.5 times the inter‐quartile range from the upper (resp. lower) limit of the box. Points with values beyond the end of the whiskers are outliers and are plotted as circles.

**Figure S11.** Canonical Correlation Analysis (CCA) using the ${\mathrm{RA}}_{\mathrm{ASV}}^{\text{Fungi}}$ of all fungal ASVs as response variables and environmental variables as explanatory variables using the samples from (A) the > 3 μm size fraction and (C) the 0.2–3 μm size fraction. (B) and (D) are respectively identical to (A) and (C), except that only the 41 major fungal ASVs are displayed for clarity. Only samples with at least 50 fungal reads were considered in calculations. Blue arrows denote the explanatory variables and red dots the response variables. The projection uses scaling 2: the angle between any couple of variables (regardless of being response or explanatory) reflect their correlation. T: Temperature. S: Salinity. O: Dissolved oxygen. CHLA: Chlorophyll a concentration. Baillaury height: height of La Baillaury River ~3.2 km south‐west of SOLA.

**Figure S12.** Correlation (Pearson’s r) of all environmental variables, the biological variables, the first 10 dimensions of the PCA computed in Figure 2 and the relative abundance of Fungi, of fungal phyla, and of the 41 fungal ASVs that gather more than 90% of the cumulative relative abundance of Fungi. The colour denotes the correlation coefficient of the two variables. Grey tiles indicate the inability to calculate a correlation coefficient for these pairs of variables. The text in tiles indicates the correlation coefficient (r) and the associated *p* value (P) if the *p* value is significant (≤ 0.05). T: Temperature. S: Salinity. O: Dissolved oxygen. NH4: NH_4_^+^ concentration. NO3: NO_3_^−^ concentration. NO2: NO_2_^−^ concentration. PO4: PO_4_^3−^ concentration. SIO4: (SiO_4_)^4−^ concentration. Baillaury height: the height of La Baillaury River ~3.2 km south‐west of SOLA. RR: Daily rainfall at Cape Béar. FFM: Average daily wind speed at 10 m at Cape Béar. Turbidity 3 m: Turbidity at 3 m below surface. Turbidity 20 m: Turbidity at 20 m below surface (~6 m above seafloor). CHLA: Chlorophyll *a* concentration. PROC, SYNC, PICOEC, NANOEC and CRYC indicate cell counts for respectively *Prochlorococcus* sp., *Synechococcus* sp., Pico‐eukaryotes, Nano‐eukaryotes and Cryptophyceae. Fungal group names (e.g., ‘Fungi’, ‘Ascomycota’, ‘ASV224’) refer to the relative abundance of each of these groups amongst eukaryotes.

**Table S1.** Characteristics of the 41 fungal ASVs representing 90% of the cumulative relative abundance of Fungi amongst eukaryotes. Columns ‘Class’, ‘Order’, ‘Family’, ‘Genus’ and ‘Species’ indicate the taxonomy of the ASV as determined by DADA2. ‘Cumul. Rel. Ab.’, ‘Cum. Rel. Ab. > 3 μm’ and ‘Cum. Rel. Ab. 0.2 – 3 μm’ represent ${\mathrm{CRA}}_{\mathrm{ASV}}^{\text{Fungi}}$ considering respectively both size fractions, only the > 3 μm size fraction, and only the 0.2–3 μm size fraction respectively. ‘Rhythmicity > 3 μm’ and ‘Rhythmicity 0.2–3 μm’ are the behaviours of the ASVs over time in the > 3 μm size fraction, and in the 0.2–3 μm size fractions respectively. They are either ‘Chaotic’, ‘Rhythmic’, ‘Ephemeral’, or a combination of two categories. Columns ‘Ter’, ‘Fre’, ‘Coa’, ‘OOS’ and ‘OOB’ indicate if we found (‘Y’) or did not find (‘N’) the ASV with a match at 100% similarity in the metaPR2 database respectively in Terrestrial samples, Freshwater samples, marine Coastal samples, Open Ocean Surface samples and Open Ocean Deep samples. * indicates that we found no sequence matching at 100% similarity in the metaPR2 database. ‘Only Med’ indicates if we found (‘Y’) the ASV only in Mediterranean waters, either at SOLA or in MetaPR2, or if the ASV was also found elsewhere (‘N’). ‘Curated taxonomy’ is the taxonomy we obtained by performing BLASTs on NCBI’s nr‐nt database. We indicate the taxonomy to the lowest taxonomic level we considered reliable. ‘Max similarity with nr‐nt’ indicates the best percent of similarity between the sequence of the ASV and a reference sequence on NCBI’s nt‐nr. We did not consider reference sequences referred to as ‘unknown eukaryote’ or ‘unknown Fungi’, and only considered sequences labelled as ‘isolate’, ‘strain’ or ‘type material’ (hence not considering ‘clones’). ‘Sequence’ indicates the sequences of the ASVs. ‘Best similarity MetaPR2’ indicates the percent of similarity between the sequence of the ASV and the best match in MetaPR2. For each environment investigated in MetaPR2 (Terrestrial, Freshwater, Coastal, Open Ocean Surface and Open Ocean Deep), four columns named ‘Best match MetaPR2’, ‘Location best match’, ‘Number samples with ASV in MetaPR2’ and ‘Total number samples in MetaPR2’ respectively indicate the maximal relative abundance of the best match to the ASV in MetaPR2 for this environment, the location of the MetaPR2 sample where this maximal relative abundance occurred, the number of samples from this environment in MetaPR2 from which we could retrieve the best match to the ASV, and the number of samples from this environment available in MetaPR2. For Open Ocean Surface and Open Ocean Deep, an additional column titled ‘Additional open ocean deep if the best location is too coastal’ indicates the most open ocean location where we retrieved the best match to the ASV for these environments, in the case we considered the ‘Location best match’ to be too coastal. The colours of the rows indicate the phylum of the ASVs in the ‘Curated taxonomy’ column (red: Ascomycota; blue: Basidiomycota; green: Chytridiomycota; orange: Cryptomycota; purple: Fungi from unknown phylum; grey: Opisthokonta from unknown kingdom).

**Table S2.** List of the direct neighbours of the 40 major fungal ASVs in the > 3 μm co‐occurrence network. The ASVs are sorted by decreasing ${\mathrm{CRA}}_{\mathrm{ASV}}^{\text{Fungi}}$. For each fungal ASVs, the direct neighbours are sorted by order of decreasing absolute weight (a measure of the strength of the association). Note that there are only 40 major fungal ASVs here as ASV971 is absent from the > 3 μm size fraction.

## Data Availability

The raw sequences were deposited in the Sequence Read Archive (SRA) of the NCBI under the accession number PRJNA579489 for the 0.2–3 μm size fraction (https://www.ncbi.nlm.nih.gov/sra/PRJNA579489) and under the accession number PRJNA1183754 for the > 3 μm size fraction (https://www.ncbi.nlm.nih.gov/sra/PRJNA1183754). All bash and R codes necessary to reproduce our analyses are available at https://github.com/EmileLaymand/MetabarcodingSOLAFungi/. The associated metadata are available from the websites of SOMLIT (https://www.somlit.fr/), Météo‐France (https://portail‐api.meteofrance.fr/web/en/api/DonneesPubliquesClimatologie) and HydroPortail (https://www.hydro.eaufrance.fr/stationhydro/Y010522001/fiche).
