# Supplementary figures and images for: Five‐Year Time Series Reveals Short‐Term Blooms of Planktonic Fungi in a Coastal Mediterranean Site
Source: Environ Microbiol Rep. 2025 Jul 12;17(4):e70154. doi: 10.1111/1758-2229.70154 (PMC12254913; doi:10.1111/1758-2229.70154)

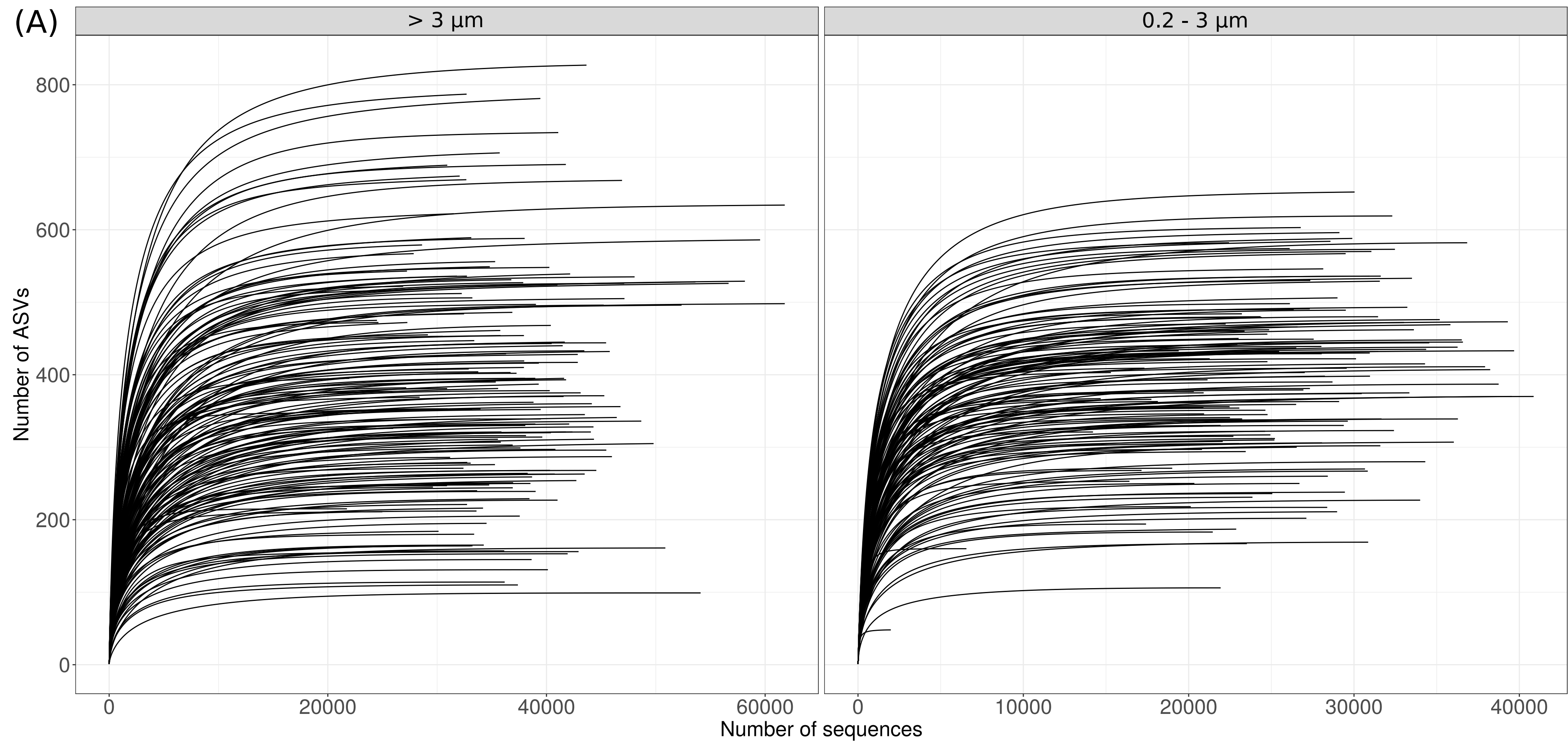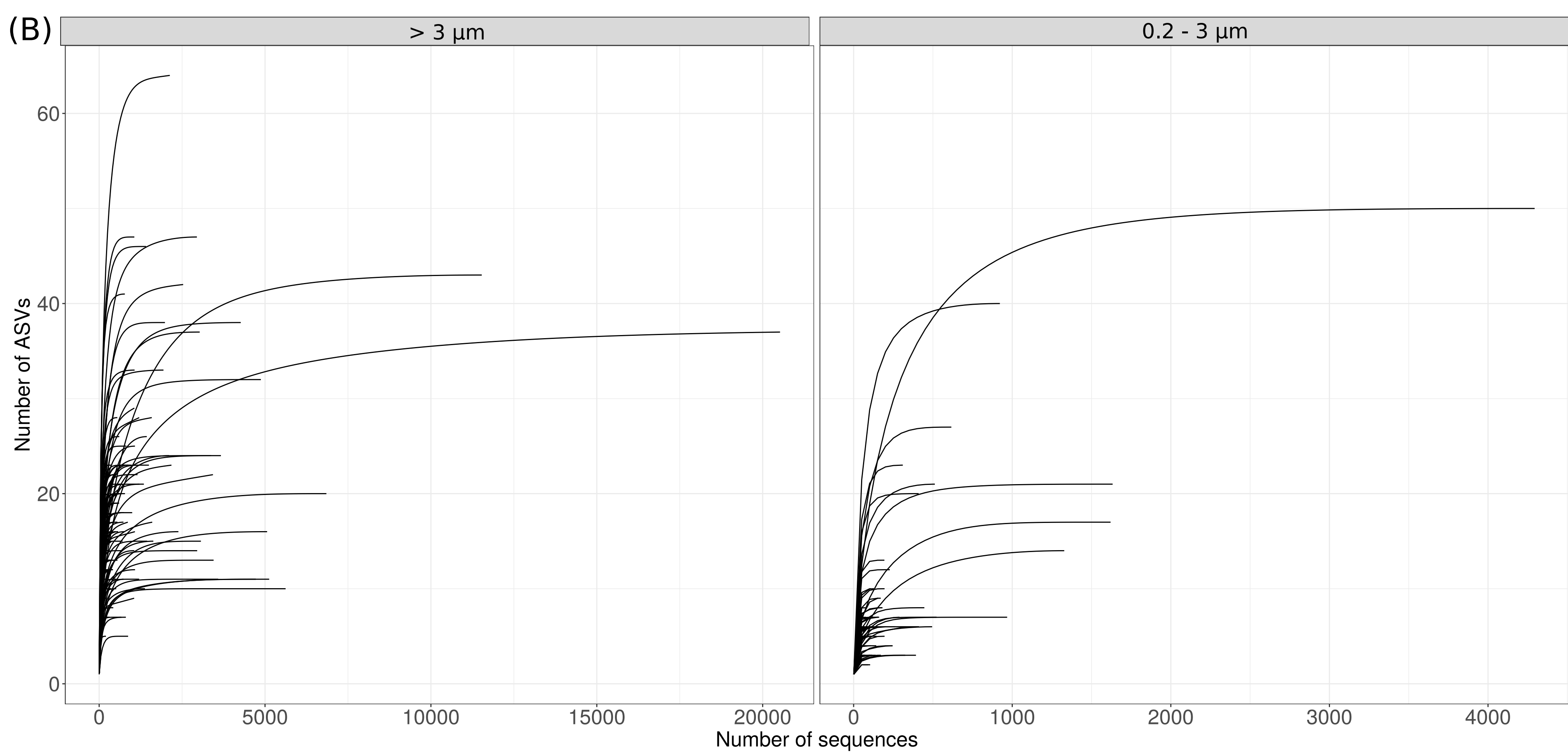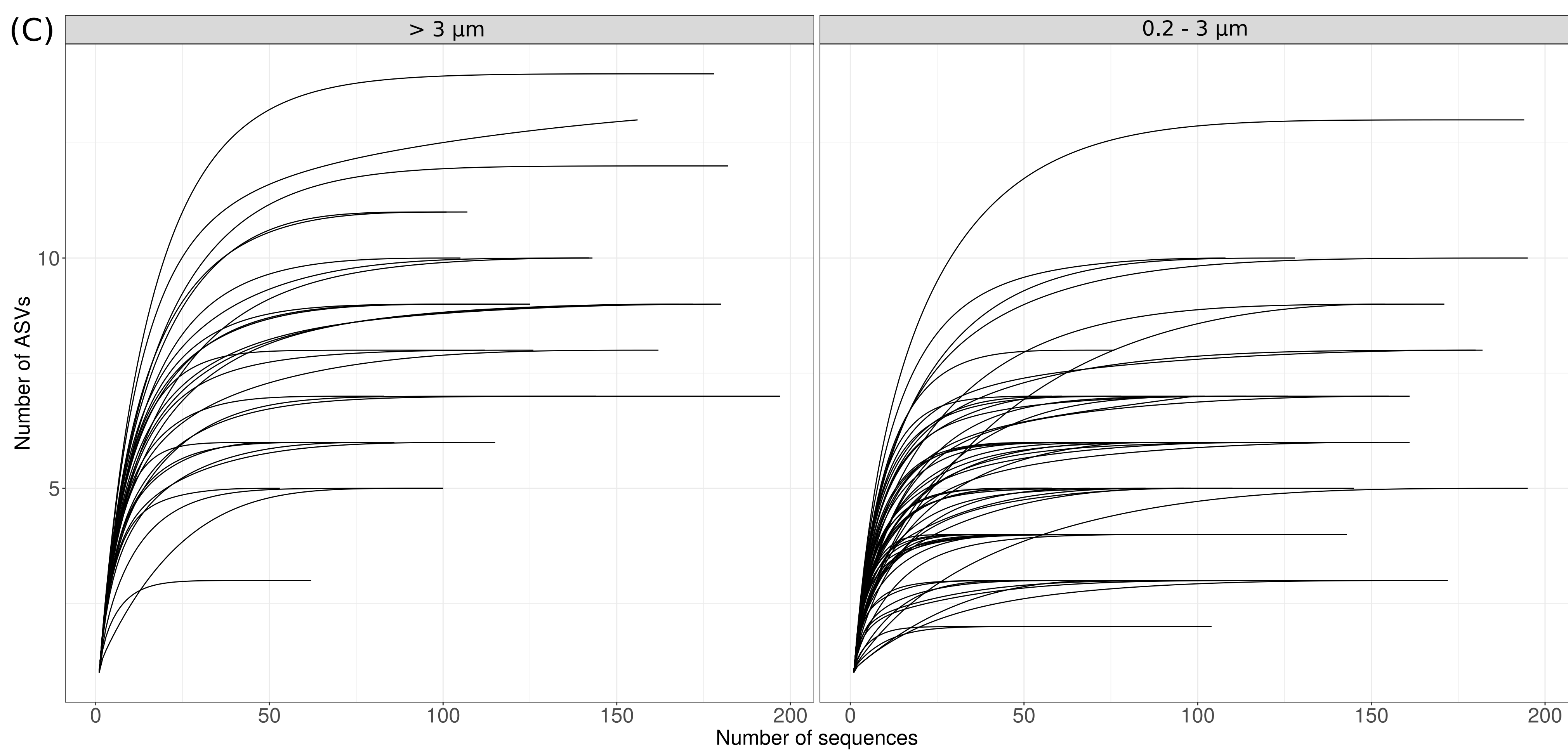

Supplement: Supplementary file 1 — Figure S1. Rarefaction curves (A) All reads—not only the reads classified as Fungi—are considered. All samples are displayed. (B) Fungal reads only. Only samples with more than 50 fungal reads are displayed. (C) Fungal reads only. Only samples that contain between 50 and 200 fungal reads are displayed. Left panels: > 3 μm size fraction. Right panels: 0.2–3 μm size fraction. [file EMI4-17-e70154-s013.pdf]

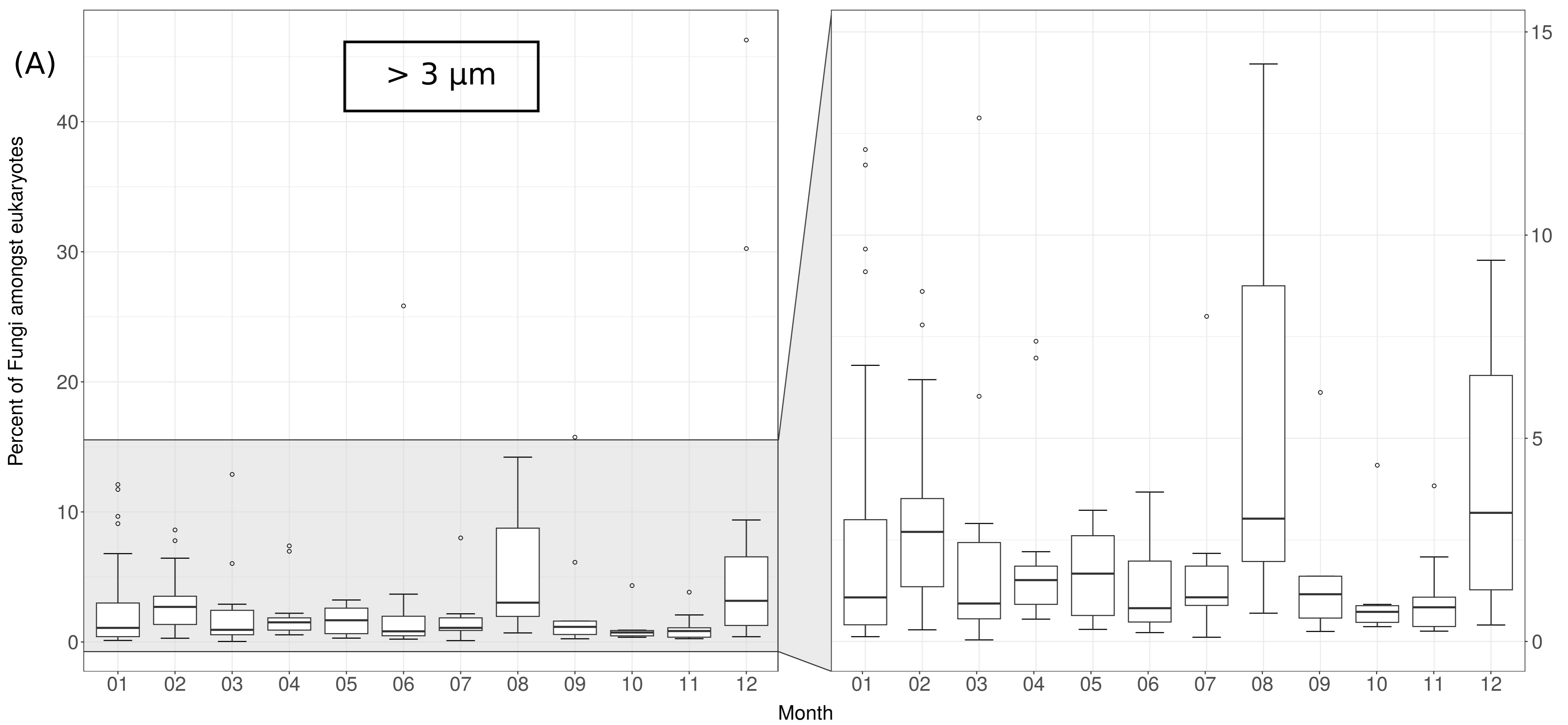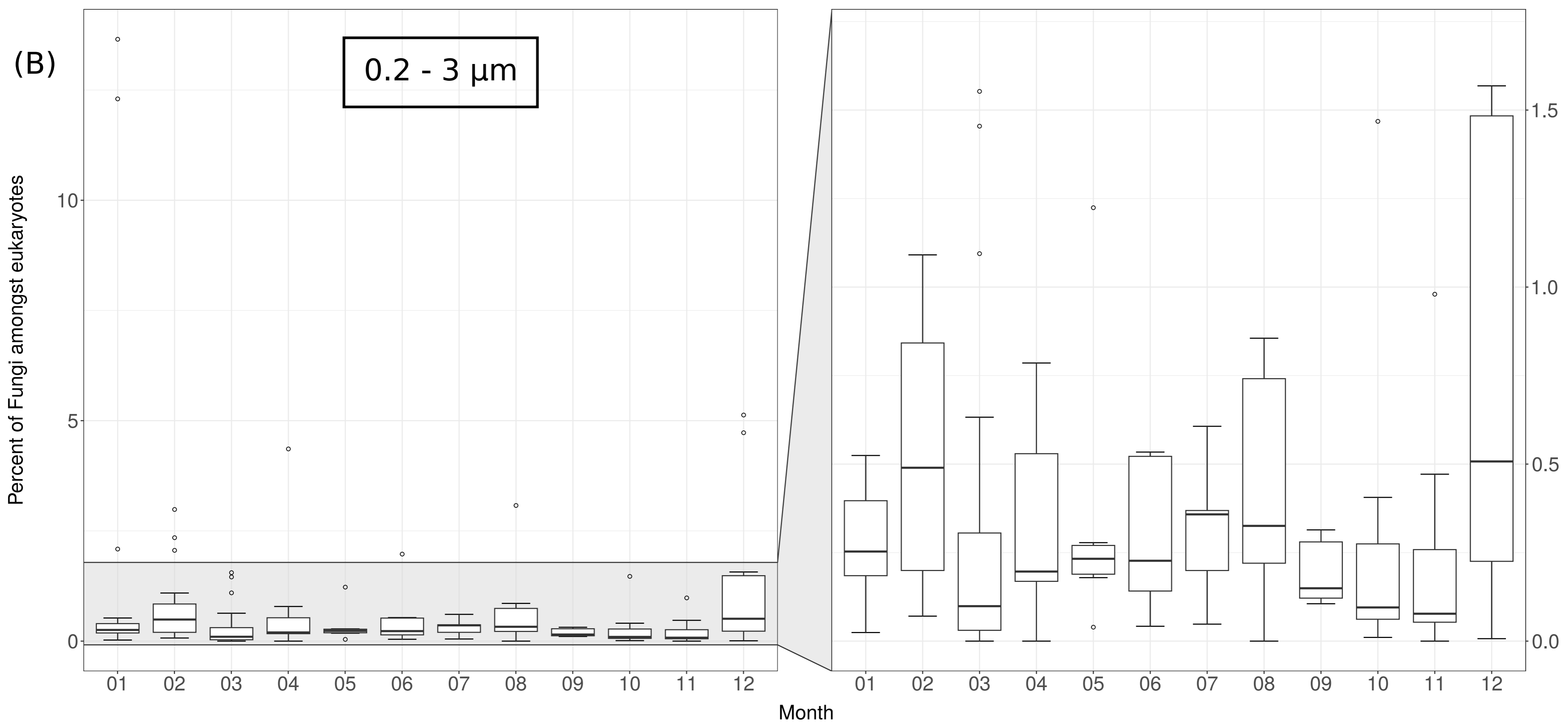

Supplement: Supplementary file 2 — Figure S2. Percent of Fungi amongst eukaryotes in function of the month of sampling. (A) > 3 μm size fraction. (B) 0.2–3 μm size fraction. Please note the difference of the y‐axis scales of panels (A) and (B). The upper and lower limits of the boxes correspond to the first and third quartiles. The horizontal line in the boxes is the median. The upper (respectively lower) whisker extends from the upper (resp. lower) limit of the box to the largest (resp. smallest) value no further than 1.5 times the inter‐quartile range from the upper (resp. lower) limit of the box. Points with values beyond the end of the whiskers are outliers and are plotted as circles. [file EMI4-17-e70154-s011.pdf]

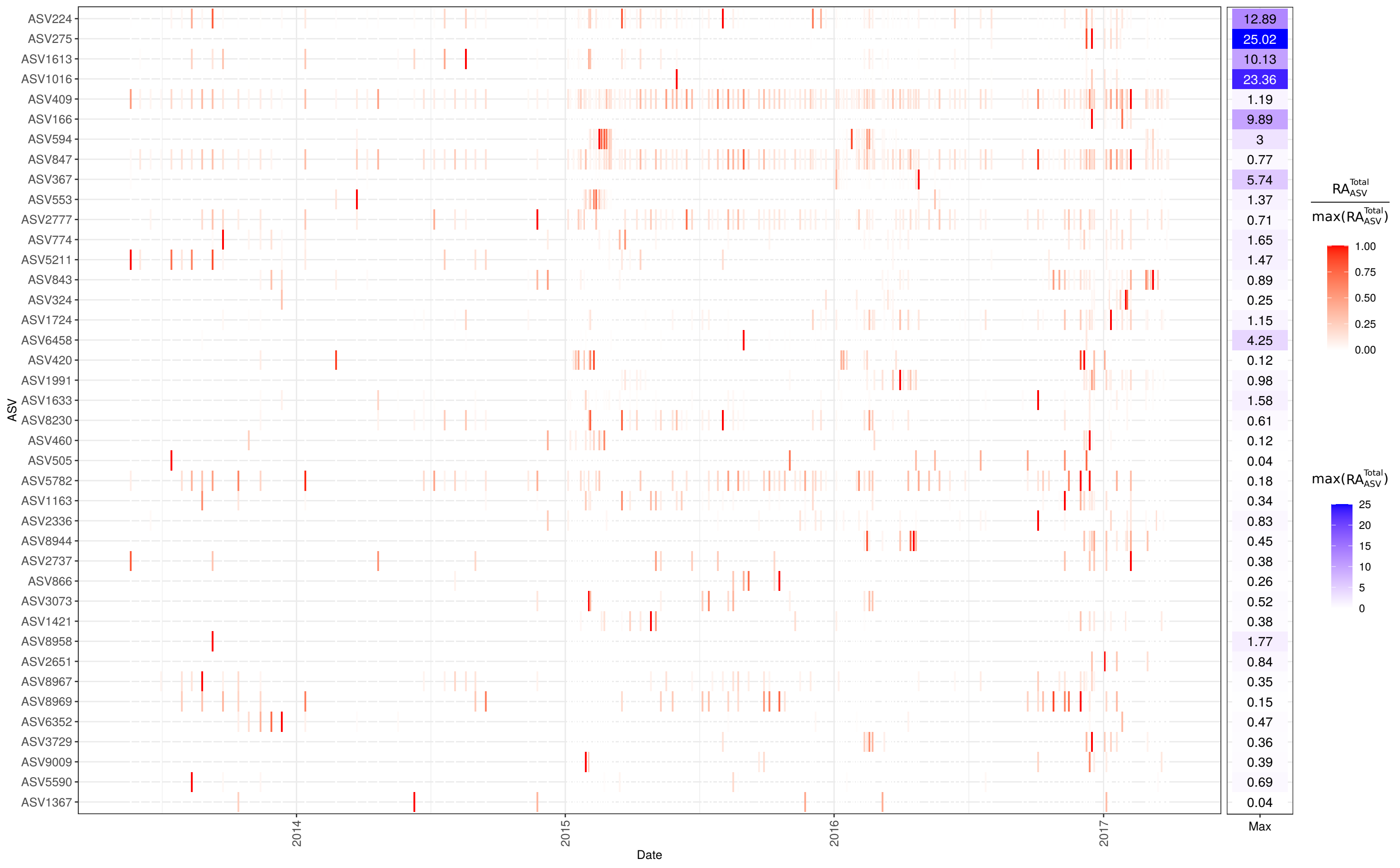

Supplement: Supplementary file 3 — Figure S3. Dynamics of the 40 major fungal ASVs present in the > 3 μm size fraction. For each ASV, the value displayed in the ‘Max’ column (purple scale) indicates the maximal RAASVTotal (noted max(RAASVTotal), in % of total reads) this ASV exhibited in this size fraction. For each ASV, we normalised every RAASVTotal value in the time series by max(RAASVTotal) in order to use the same colour scale for all ASVs. The resulting value RAASVTotal/max(RAASVTotal) is displayed with a red scale. [file EMI4-17-e70154-s001.pdf]

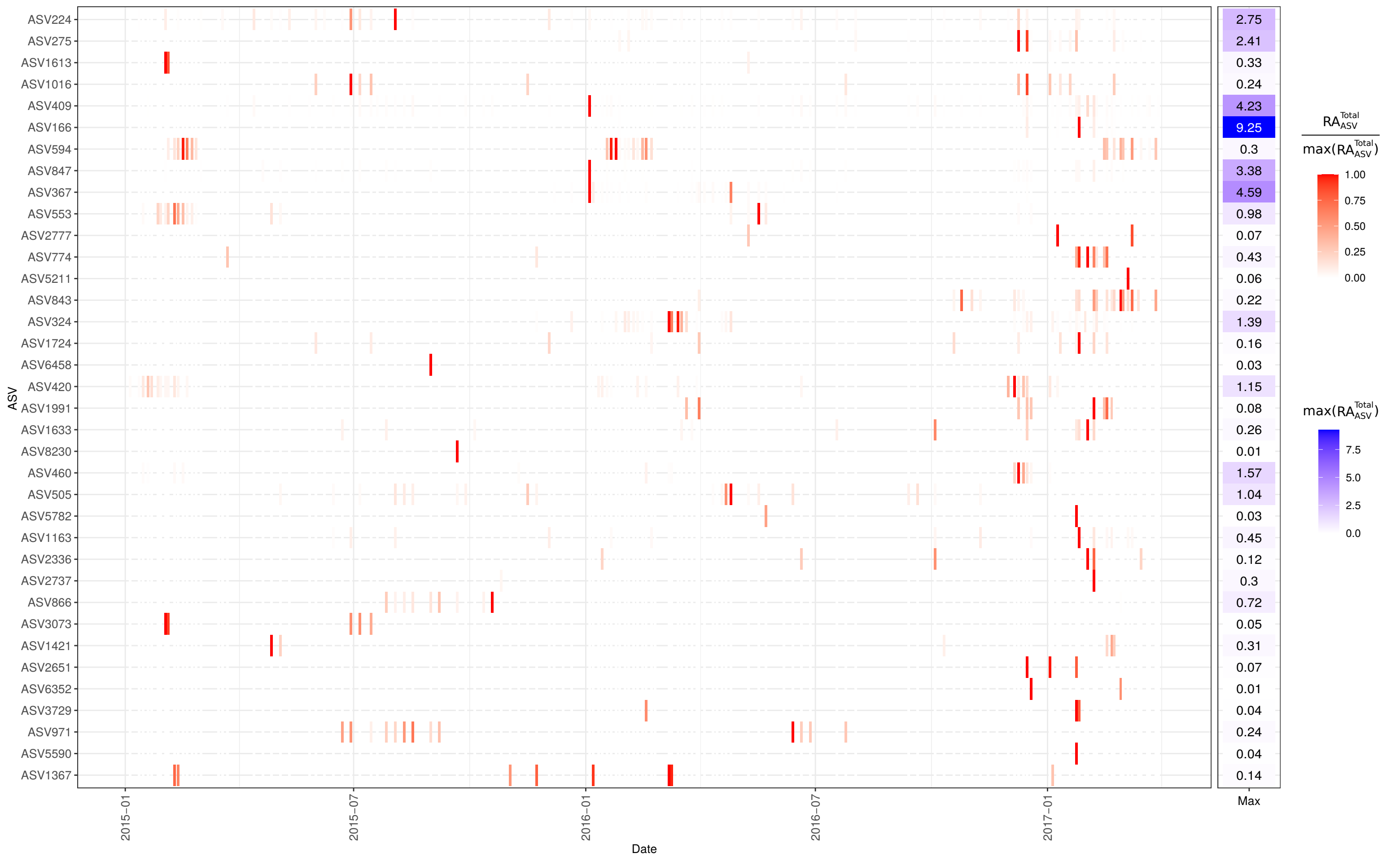

Supplement: Supplementary file 4 — Figure S4. Dynamics of the 36 major fungal ASVs present in the 0.2–3 μm size fraction. For each ASV, the value displayed in the ‘Max’ column (purple scale) indicates the maximal RAASVTotal (noted max(RAASVTotal), in % of total reads) this ASV exhibited in this size fraction. For each ASV, we normalised every RAASVTotal value in the time series by max(RAASVTotal) in order to use the same colour scale for all ASVs. The resulting value RAASVTotal/max(RAASVTotal) is displayed with a red scale. [file EMI4-17-e70154-s009.pdf]

> 3  $\mu\text{m}$

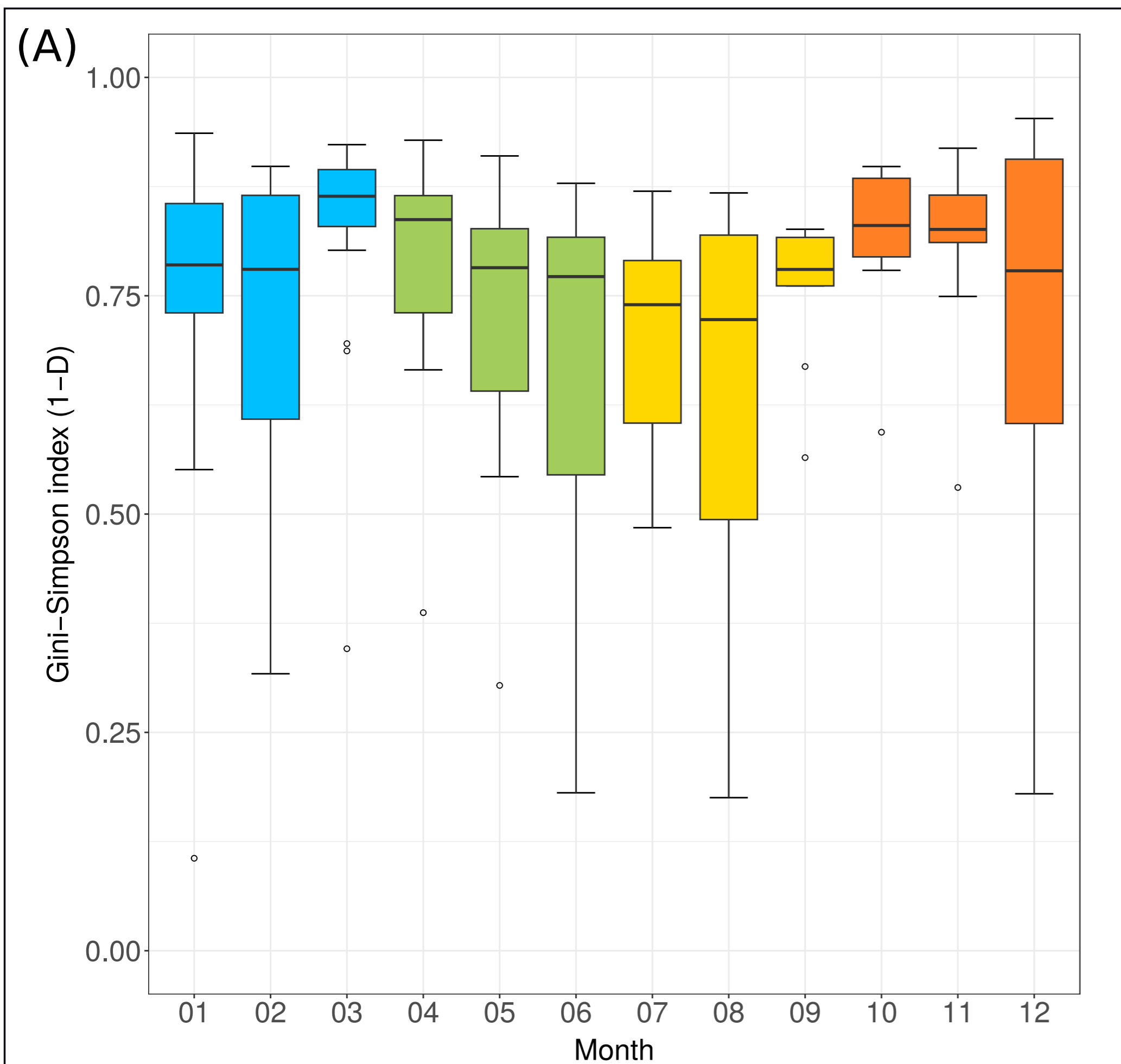

0.2 - 3  $\mu\text{m}$

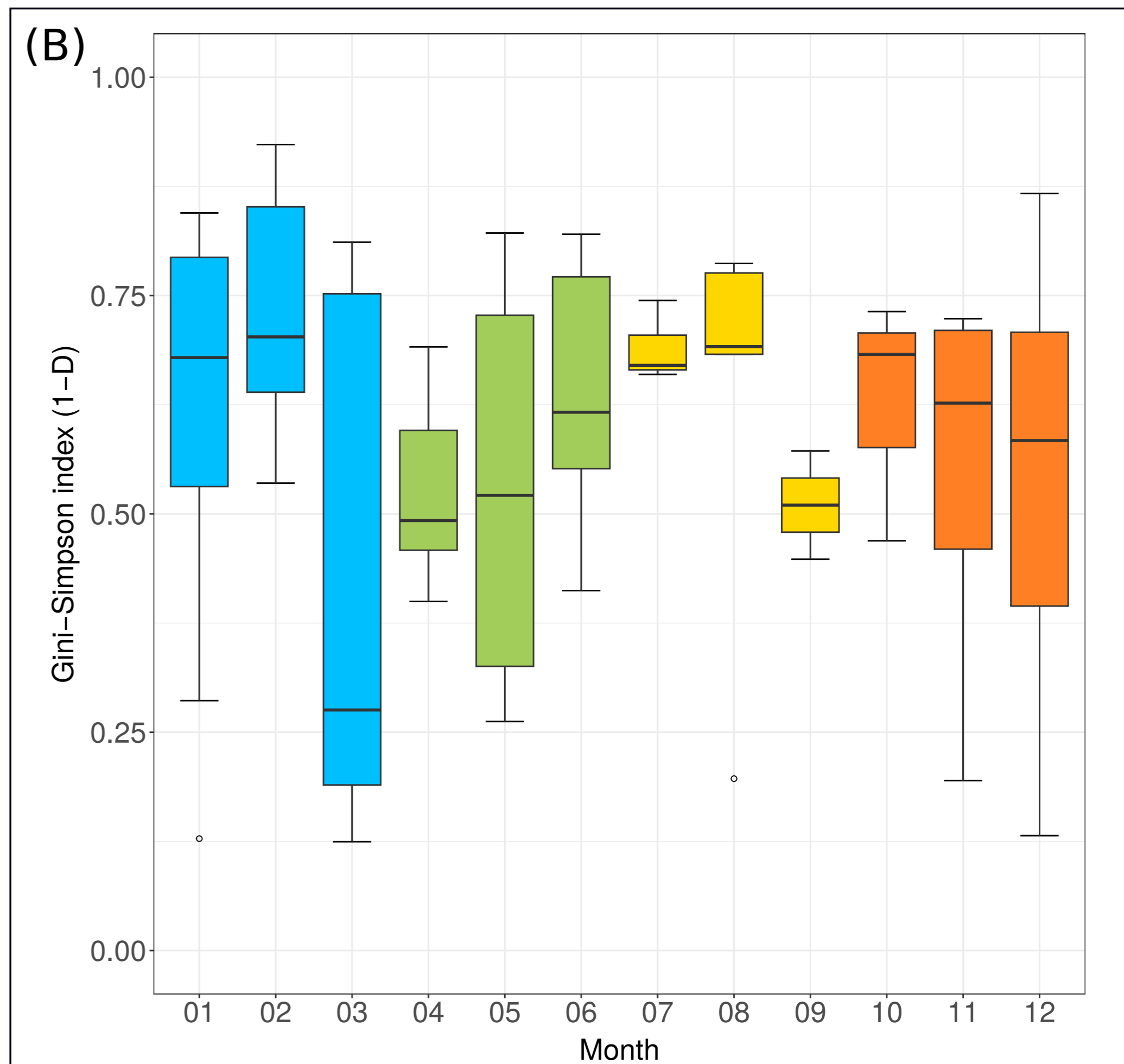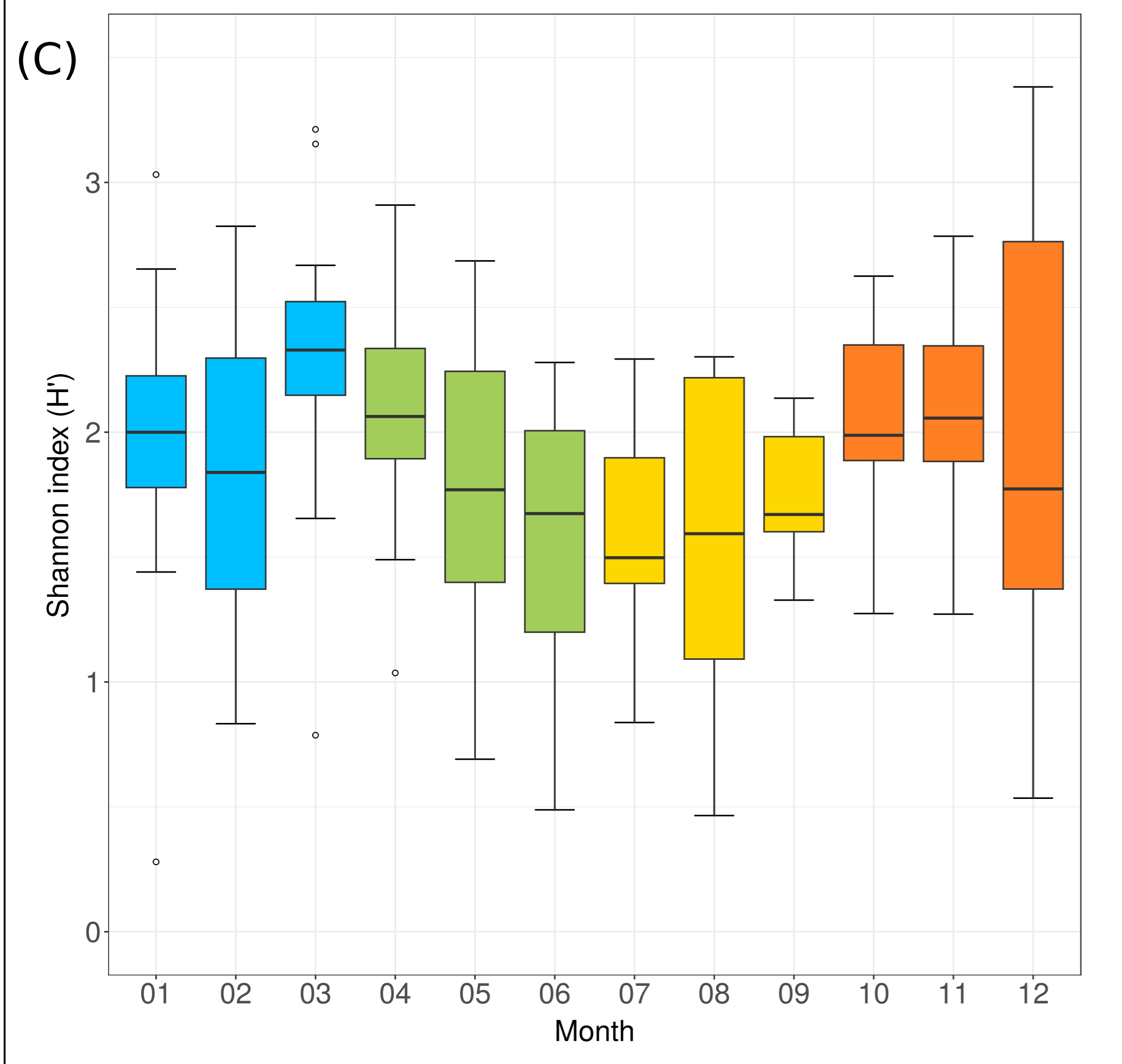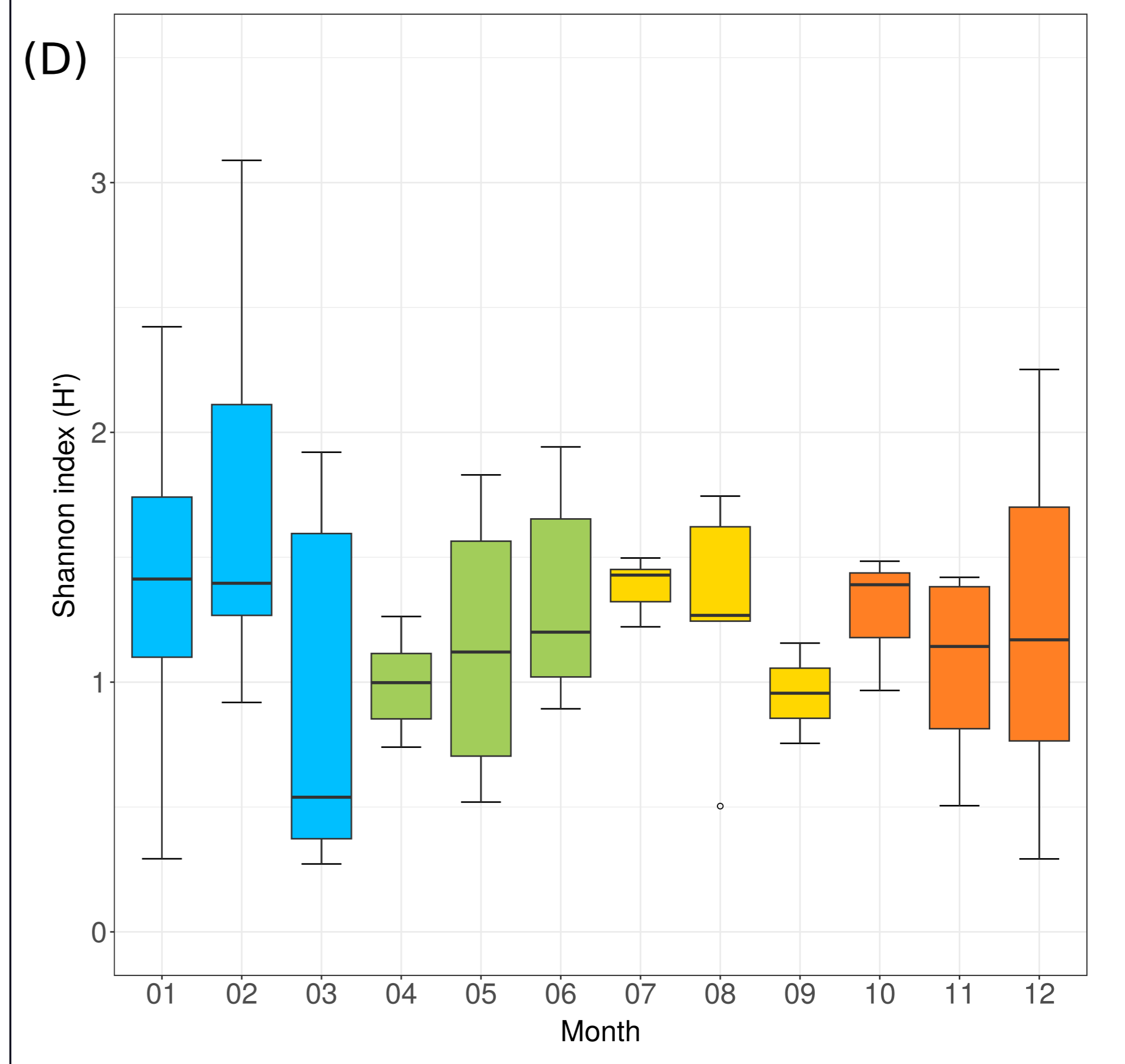

Winter Spring Summer Autumn

Supplement: Supplementary file 5 — Figure S5. Alpha diversity within kingdom Fungi in function of the month of sampling. Indices used are the Gini‐Simpson index (1 − D) in (A) the > 3 μm and (B) the 0.2–3 μm size fractions, and the Shannon index (H′) in (C) the > 3 μm and (D) the 0.2–3 μm size fractions. Colours indicate the season of sampling. Only samples with at least 50 fungal reads were considered in calculations. [file EMI4-17-e70154-s006.pdf]

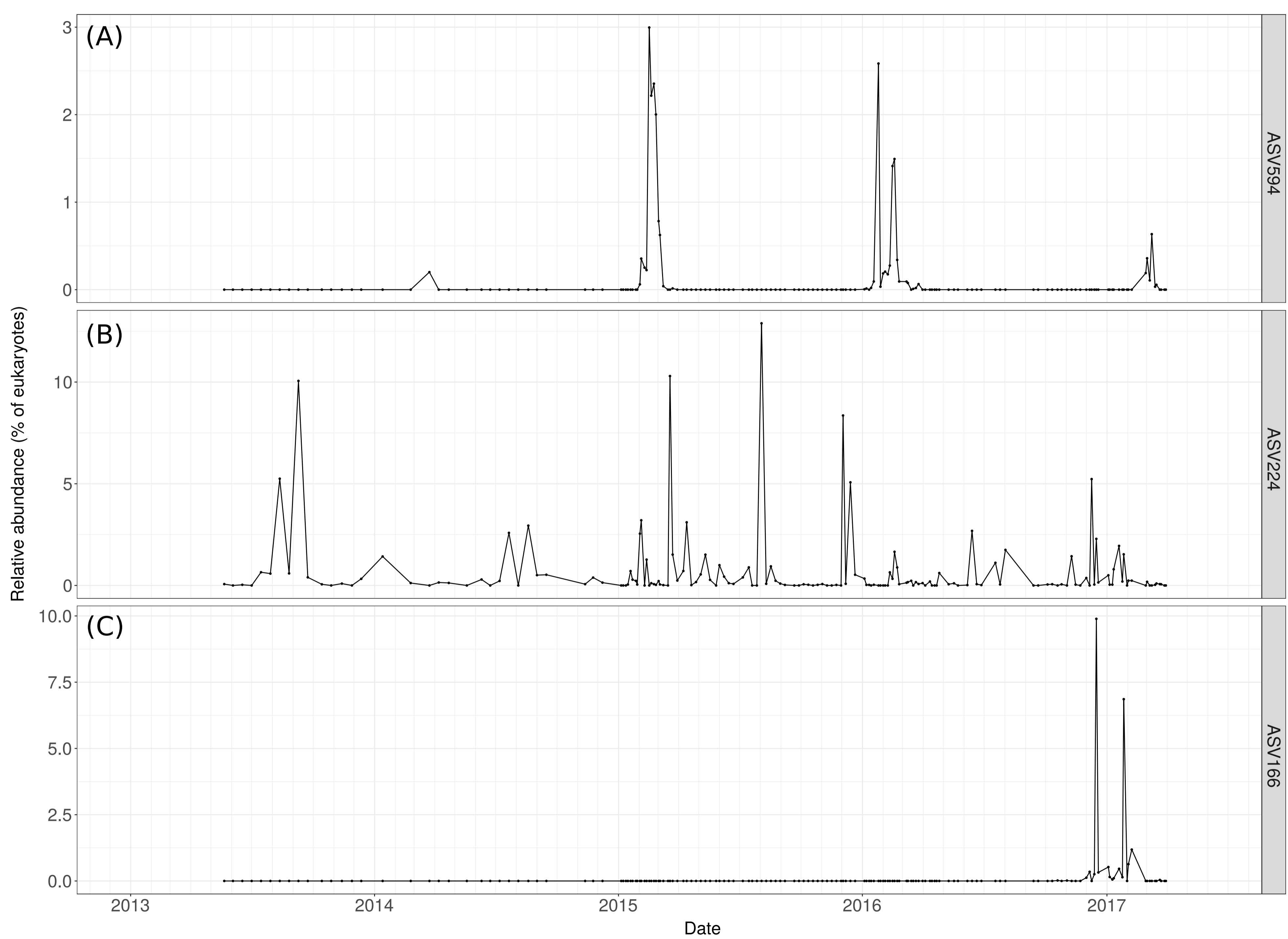

Supplement: Supplementary file 6 — Figure S6. Relative abundance amongst eukaryotes in the > 3 μm size fraction of (A) ASV594 (‘rhythmic’), (B) ASV224 (‘chaotic’) and (C) ASV166 (‘ephemeral’). [file EMI4-17-e70154-s003.pdf]

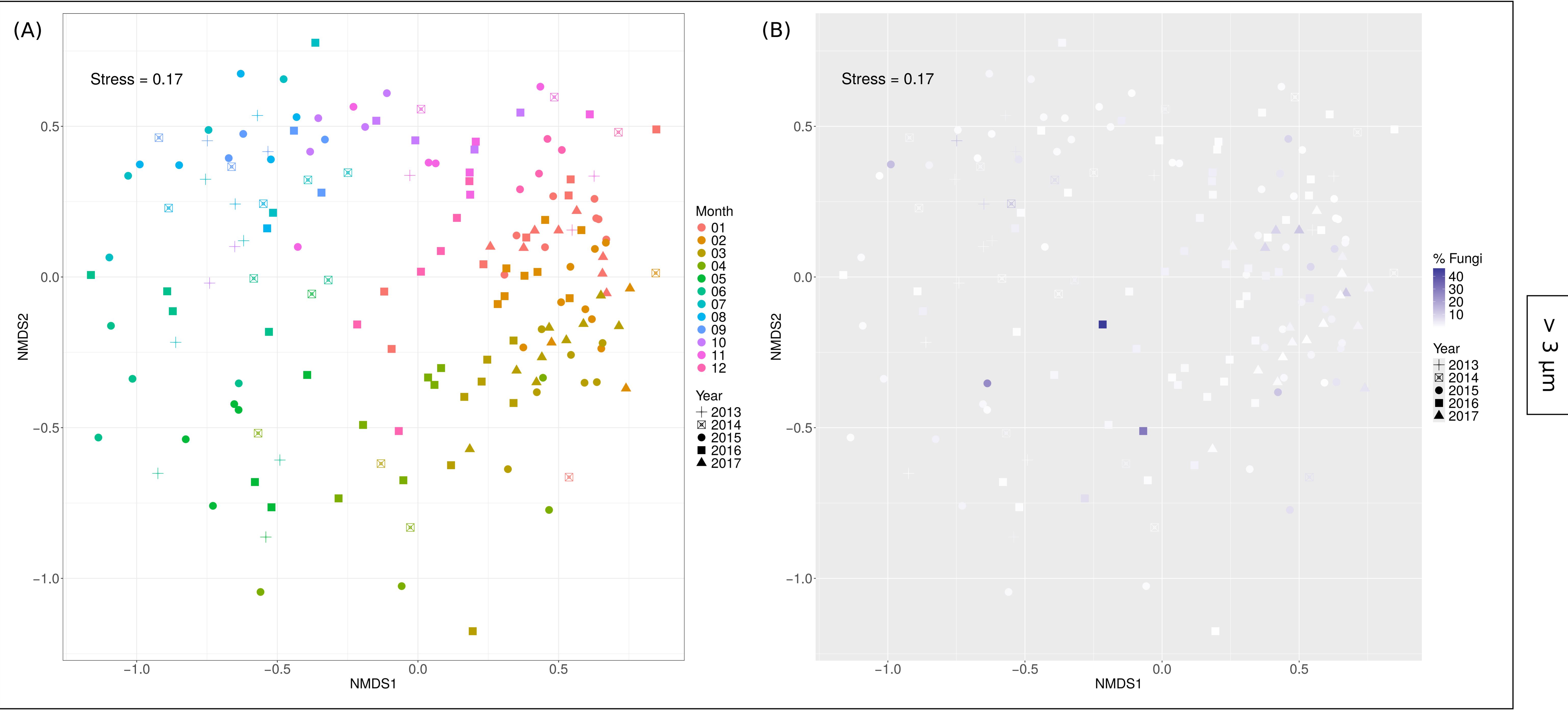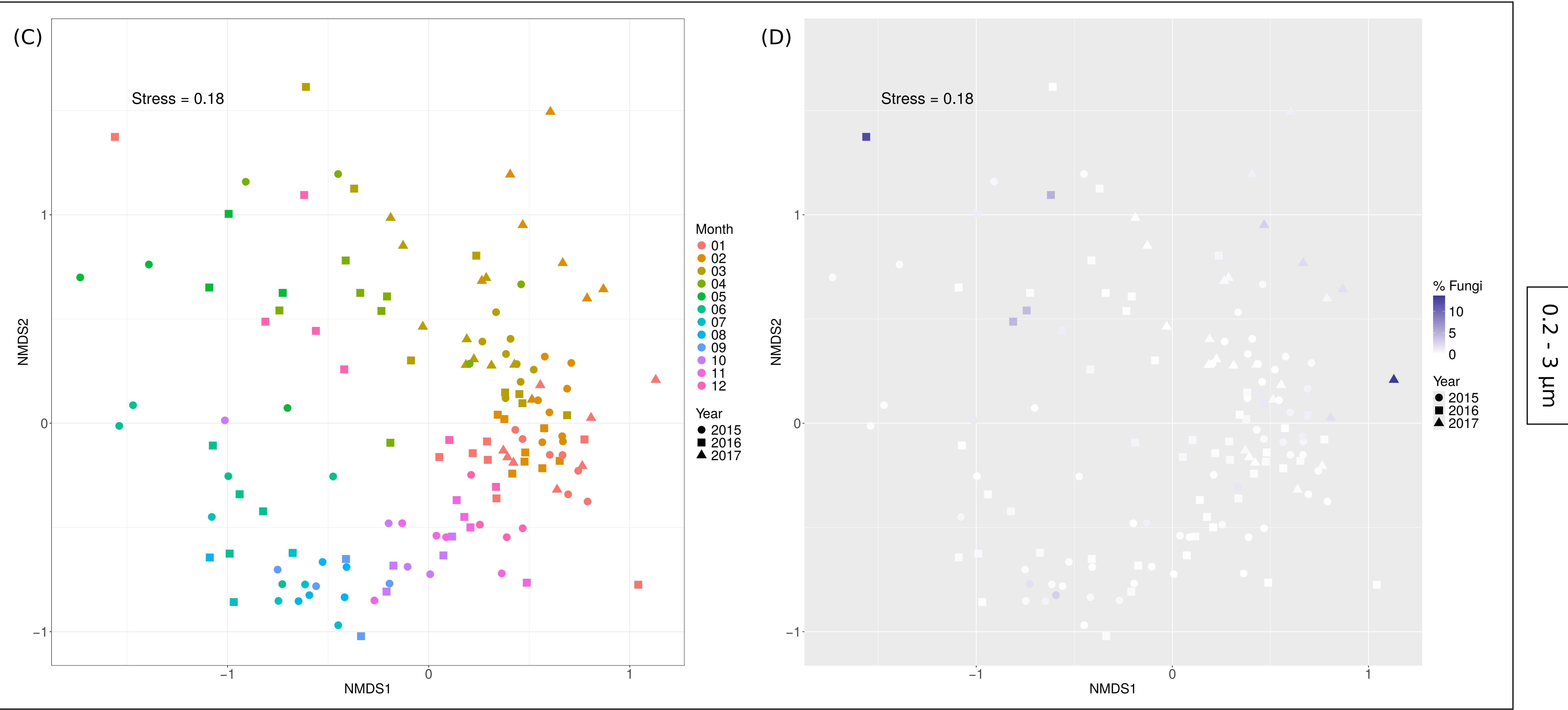

Supplement: Supplementary file 7 — Figure S7. NMDS computed with non‐fungal ASVs only, using Bray–Curtis dissimilarity directly at the ASV level. (A) and (C) Colours indicate the month in which the sample was collected, respectively for the > 3 μm size fraction and the 0.2–3 μm size fraction. (B) and (D) Colours indicate the percent of Fungi amongst eukaryotes in the sample, respectively for the > 3 μm size fraction and the 0.2–3 μm size fraction. [file EMI4-17-e70154-s008.pdf]

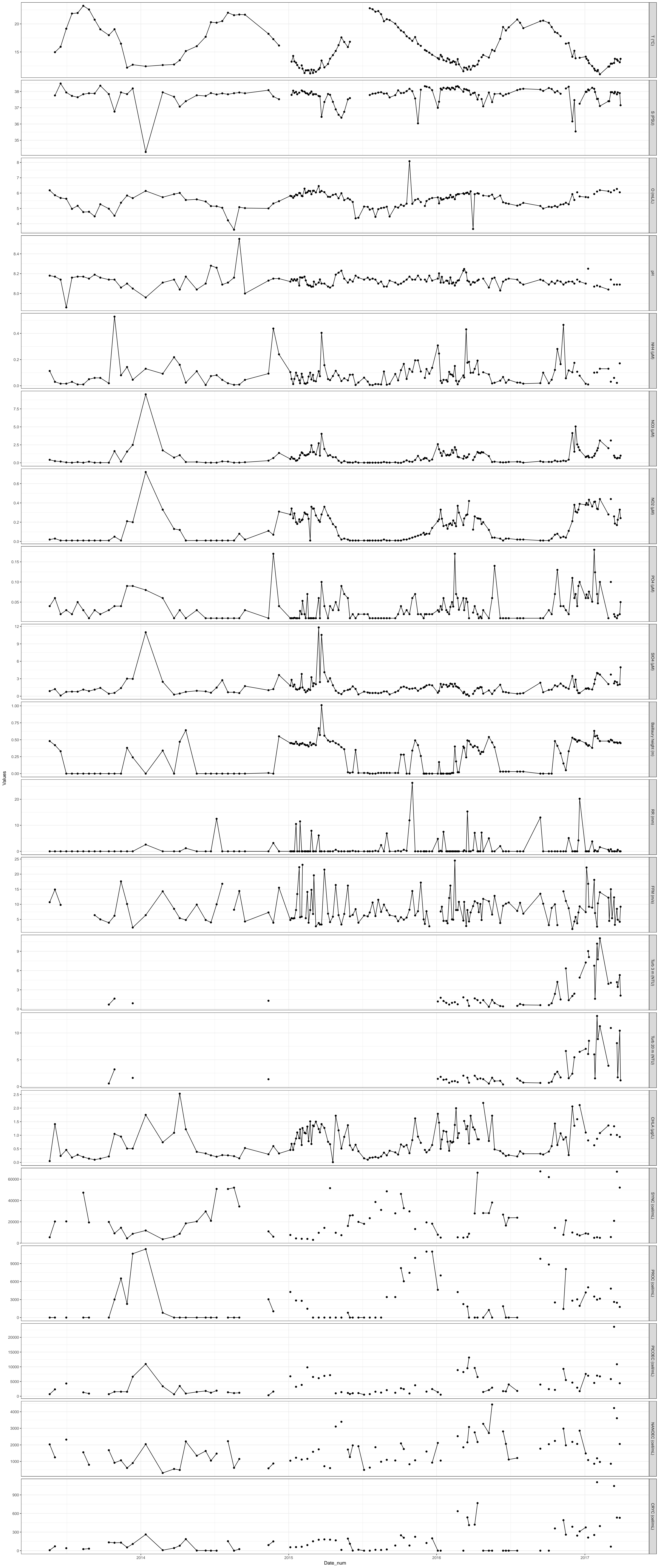

Supplement: Supplementary file 8 — Figure S8. Variations over the sampling period of the environmental parameters. (A) Temperature, (B) Salinity, (C) Dissolved oxygen, (D) pH, (E) NH4 + concentration, (F) NO3 − concentration, (G) NO2 − concentration, (H) PO4 3− concentration, (I) (SiO4)4− concentration, (J) The height of La Baillaury river ~3.2 km south‐west of SOLA, (K) Daily rainfall at Cape Béar, (L) Average daily wind speed at 10 m at Cape Béar, (M) Turbidity at 3 m below surface, (N) Turbidity at 20 m below surface (~6 m above seafloor), (O) Chlorophyll a concentration, (P) Synechococcus sp. cell count, (Q) Prochlorococcus sp. cell count, (R) Pico‐eukaryotes cell count, (S) Nano‐eukaryotes cell count, (T) Cryptophyceae cell count. [file EMI4-17-e70154-s002.pdf]

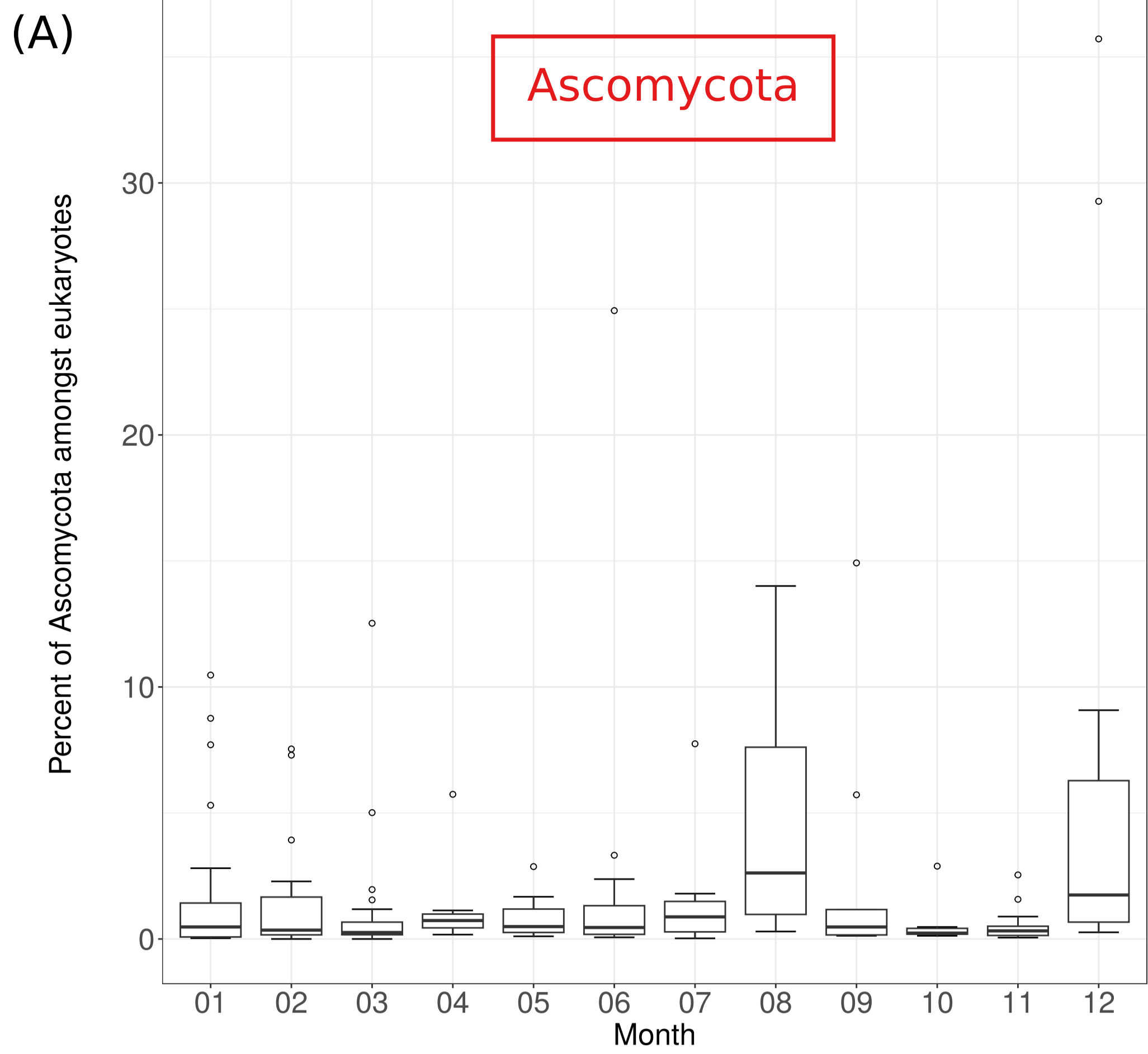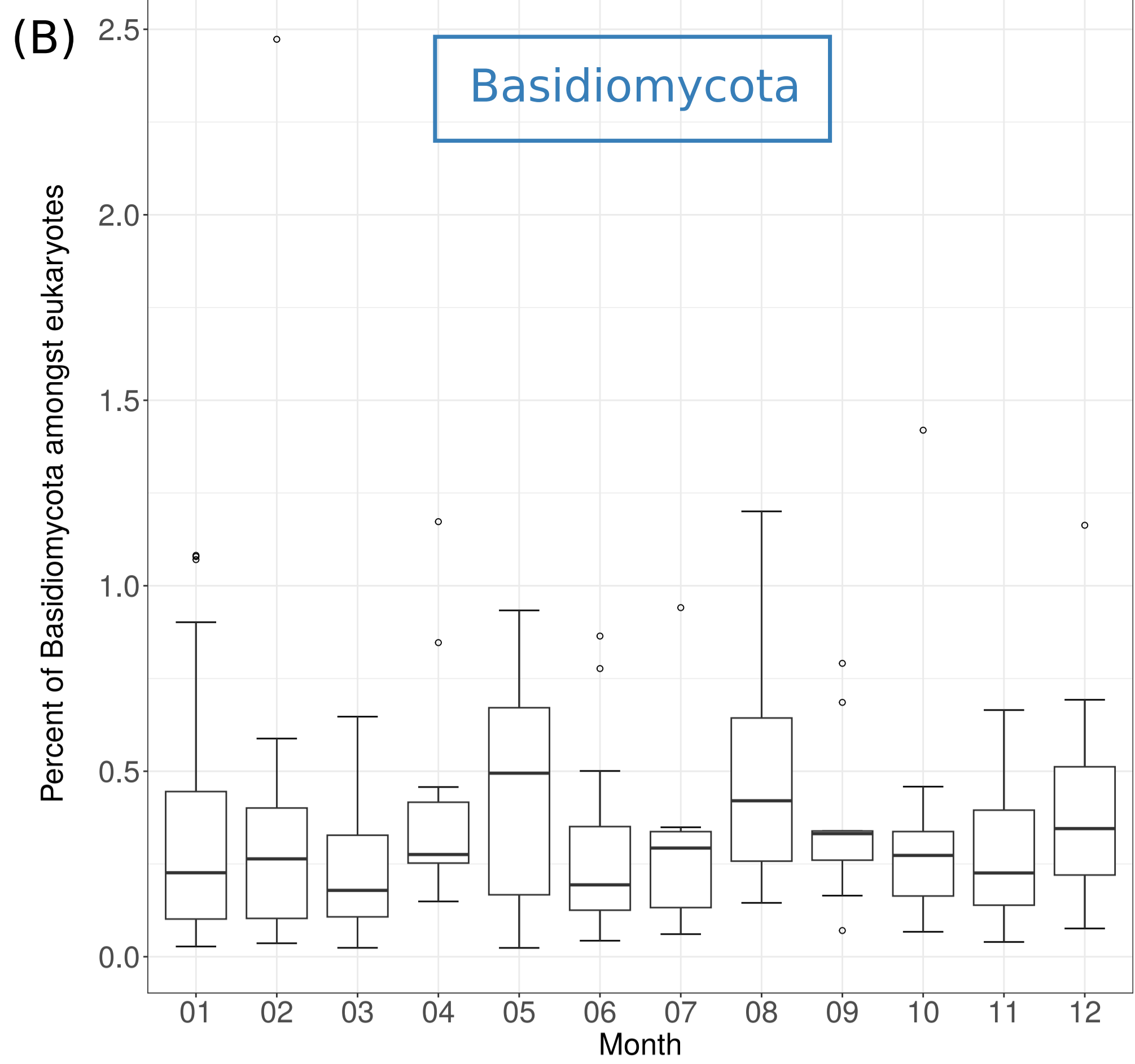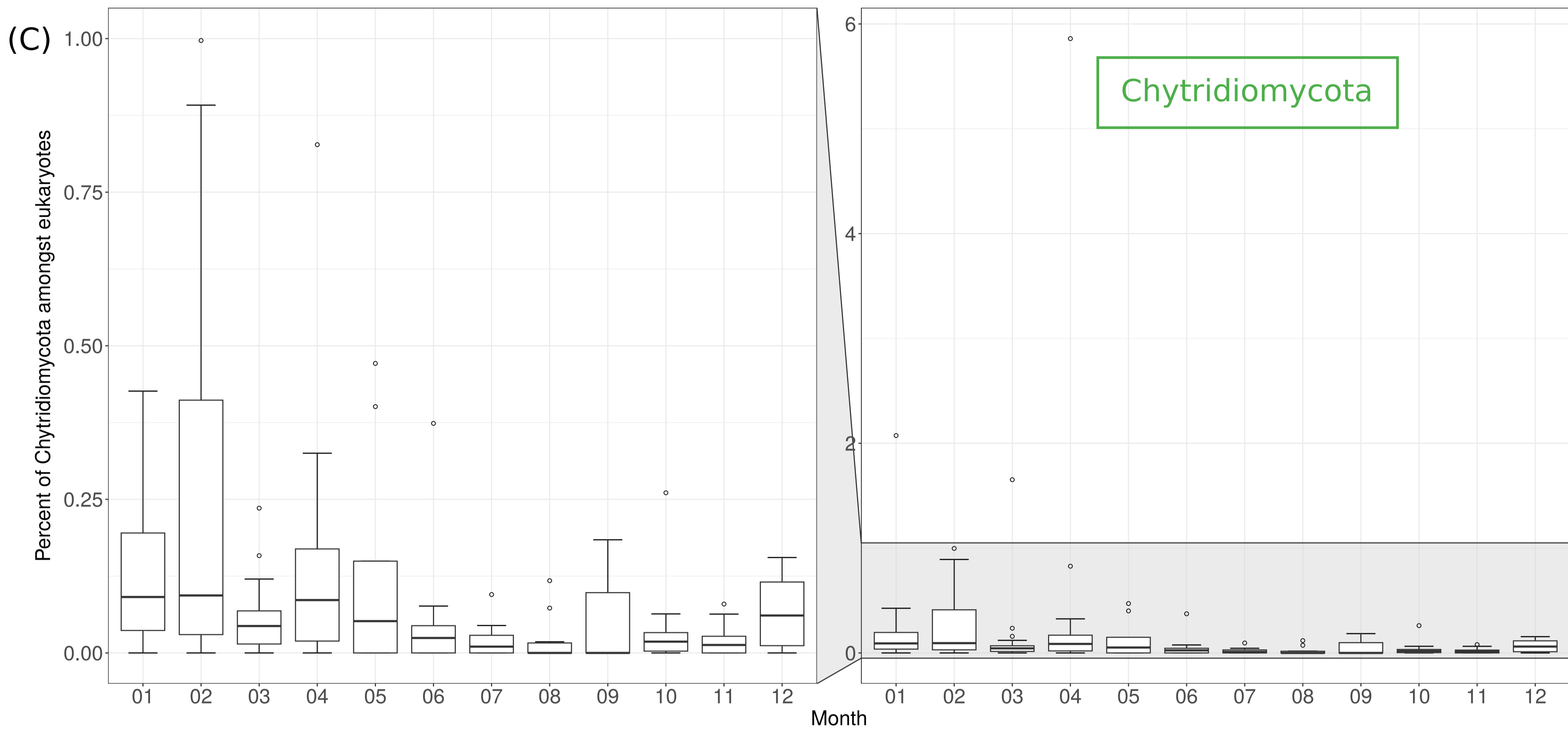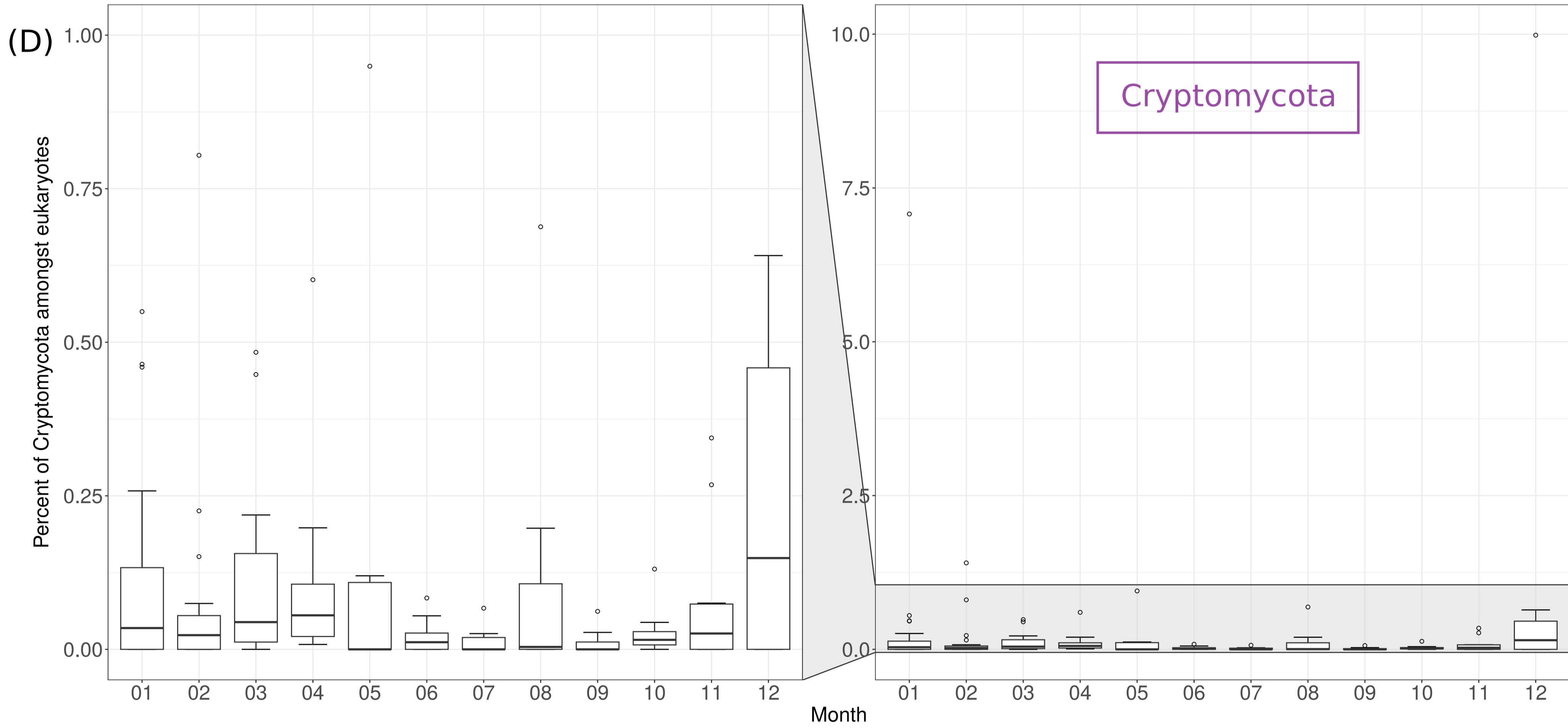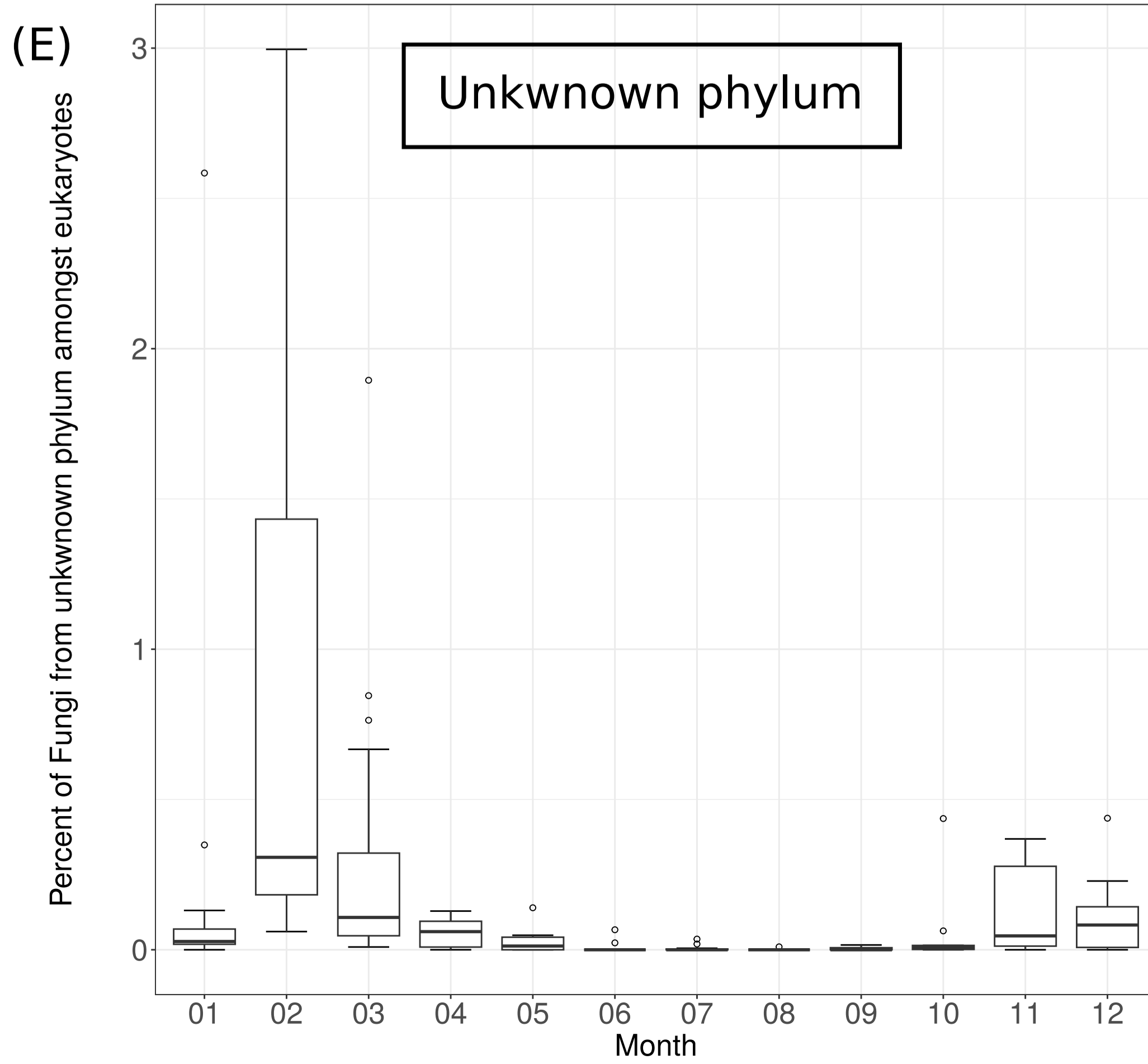

Supplement: Supplementary file 9 — Figure S9. Relative abundance amongst Eukaryotes of (A) Ascomycota, (B) Basidiomycota, (B) Chytridiomycota, (D) Cryptomycota and (E) Fungi from unknown phylum, per month over the 5 years of sampling in the > 3 μm size fraction. The upper and lower limits of the boxes correspond to the first and third quartiles. The horizontal line in the boxes is the median. The upper (respectively lower) whisker extends from the upper (resp. lower) limit of the box to the largest (resp. smallest) value no further than 1.5 times the inter‐quartile range from the upper (resp. lower) limit of the box. Points with values beyond the end of the whiskers are outliers and are plotted as circles. [file EMI4-17-e70154-s012.pdf]

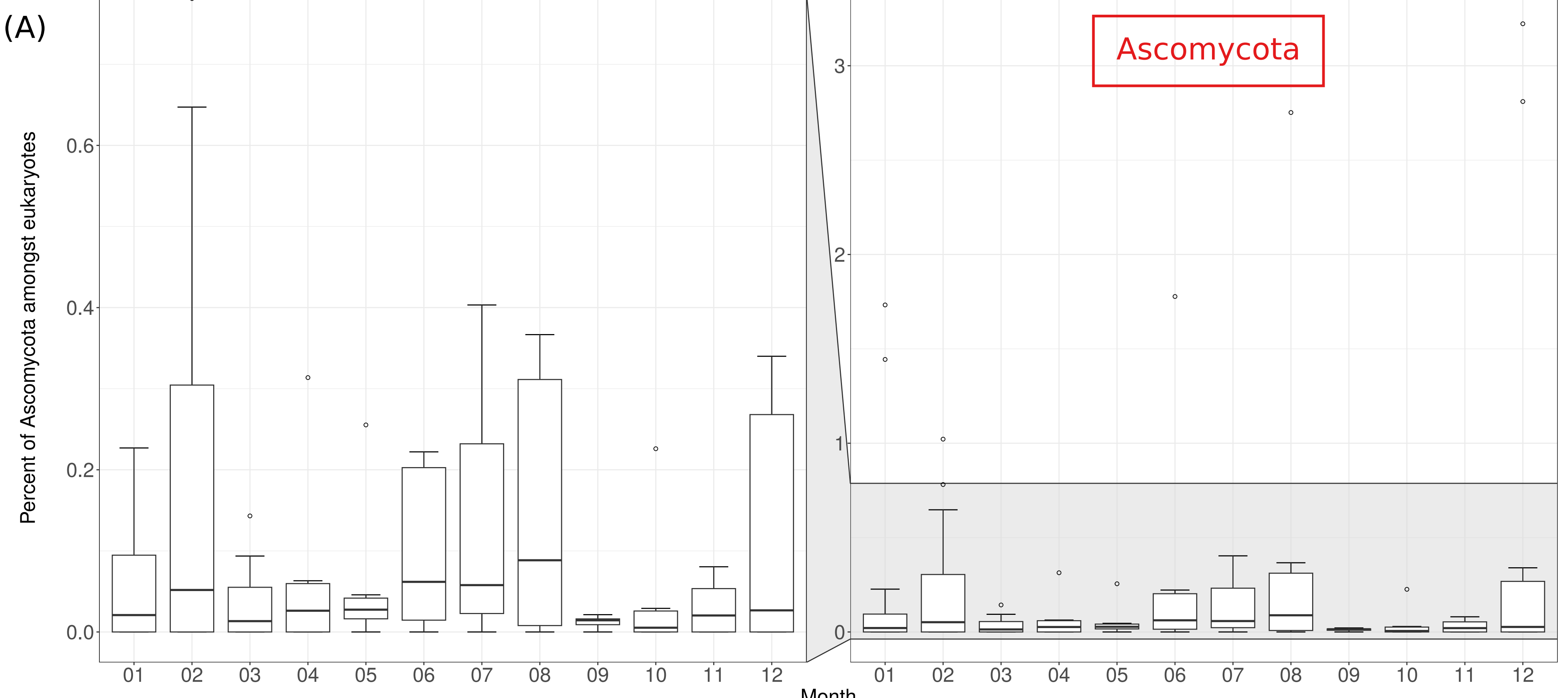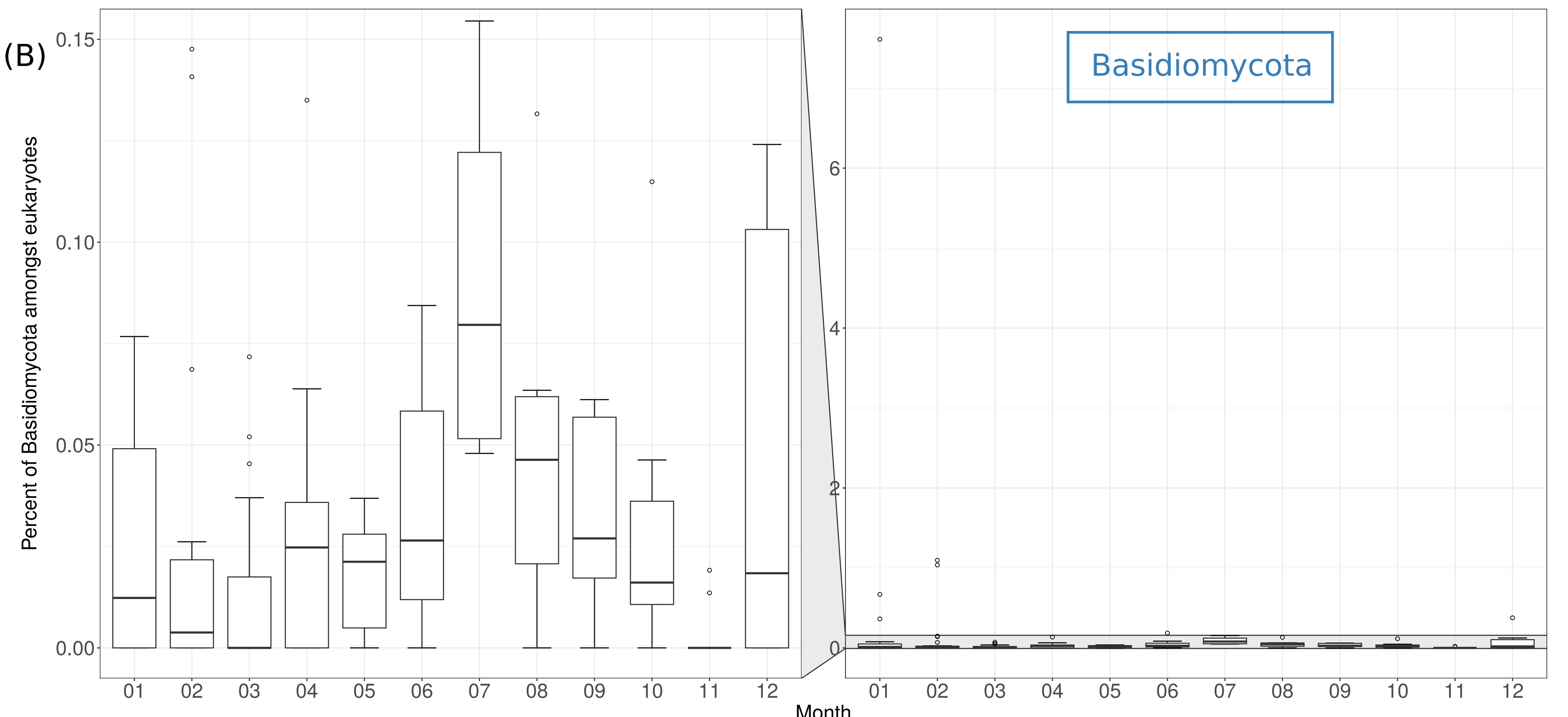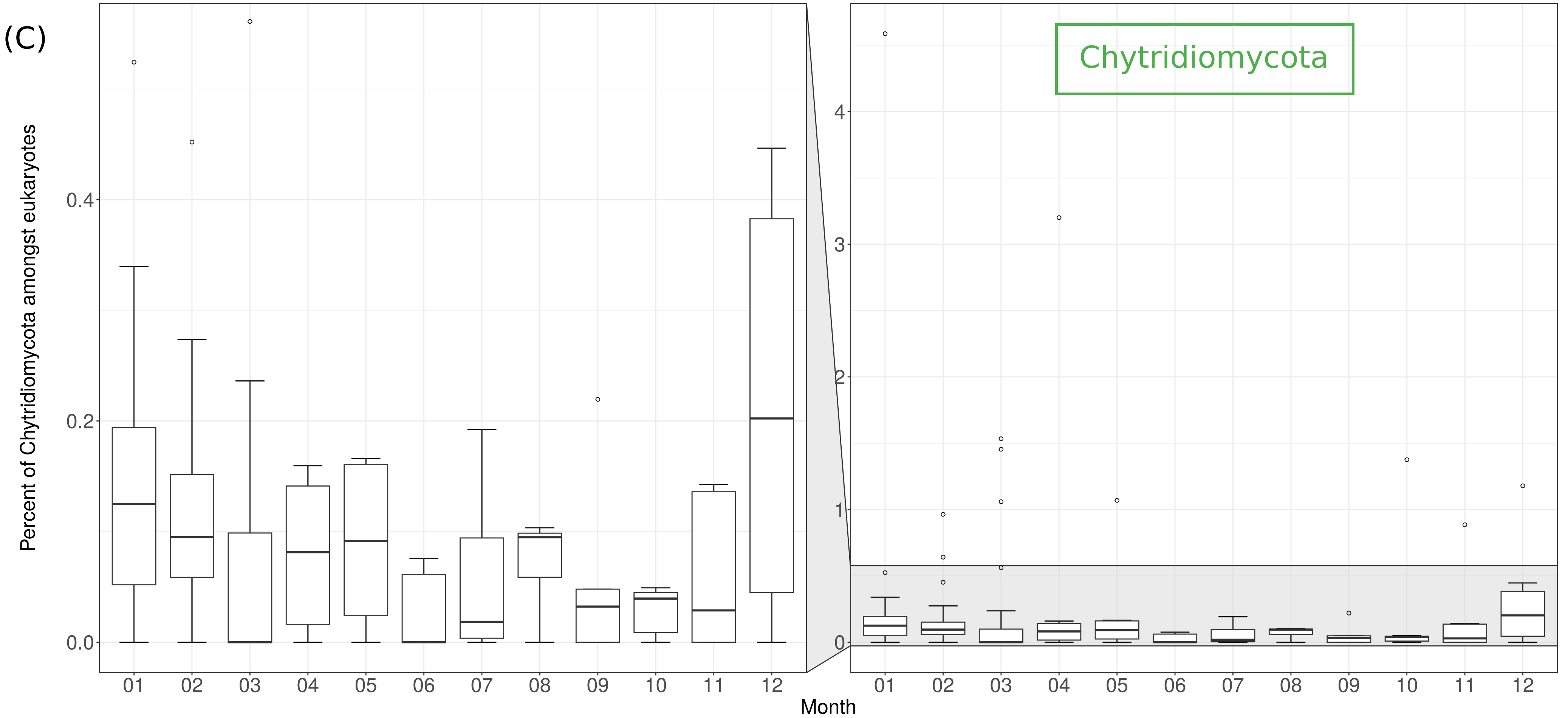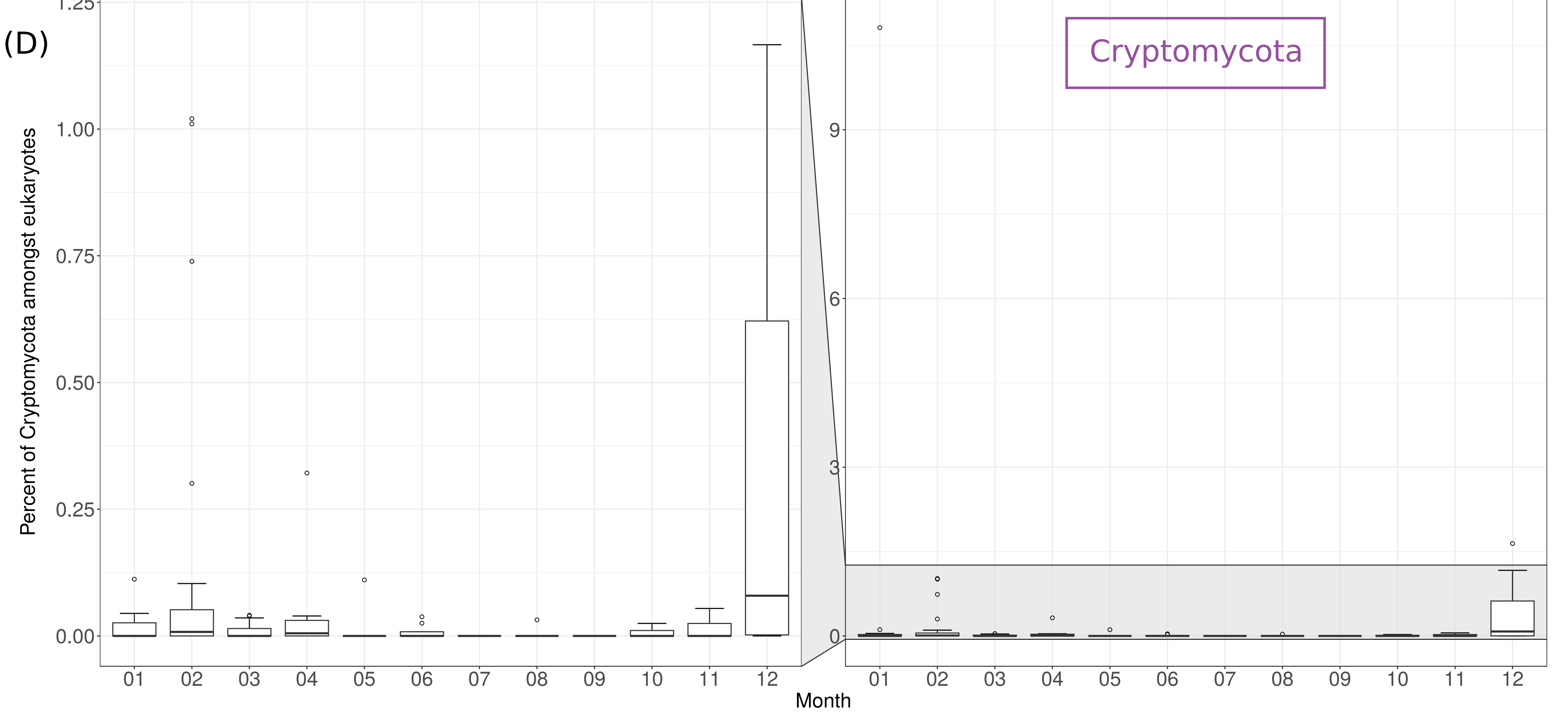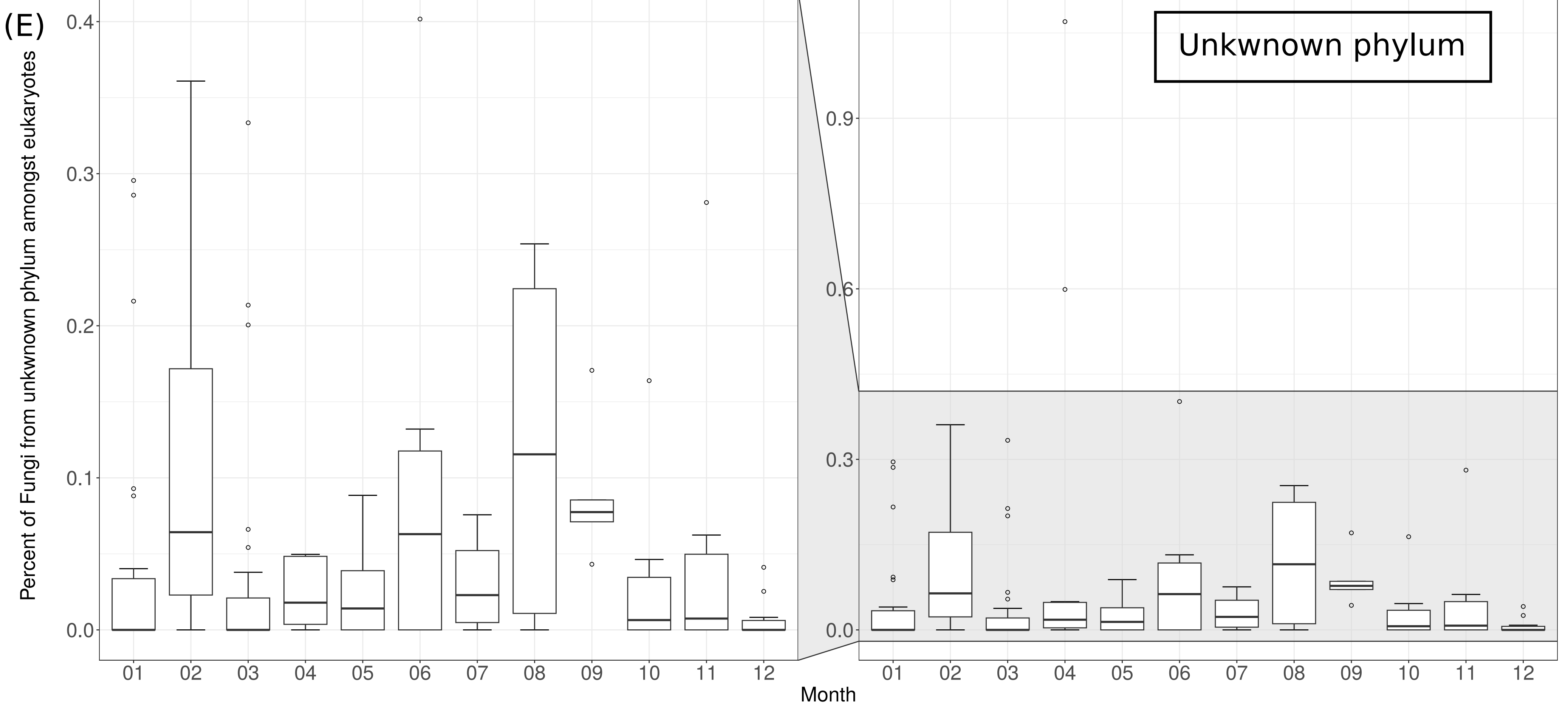

Supplement: Supplementary file 10 — Figure S10. Relative abundance amongst Eukaryotes of (A) Ascomycota, (B) Basidiomycota, (C) Chytridiomycota, (D) Cryptomycota and (E) Fungi from unknown phylum, per month over the 5 years of sampling in the 0.2–3 μm size fraction. The upper and lower limits of the boxes correspond to the first and third quartiles. The horizontal line in the boxes is the median. The upper (respectively lower) whisker extends from the upper (resp. lower) limit of the box to the largest (resp. smallest) value no further than 1.5 times the inter‐quartile range from the upper (resp. lower) limit of the box. Points with values beyond the end of the whiskers are outliers and are plotted as circles. [file EMI4-17-e70154-s005.pdf]

> 3  $\mu\text{m}$

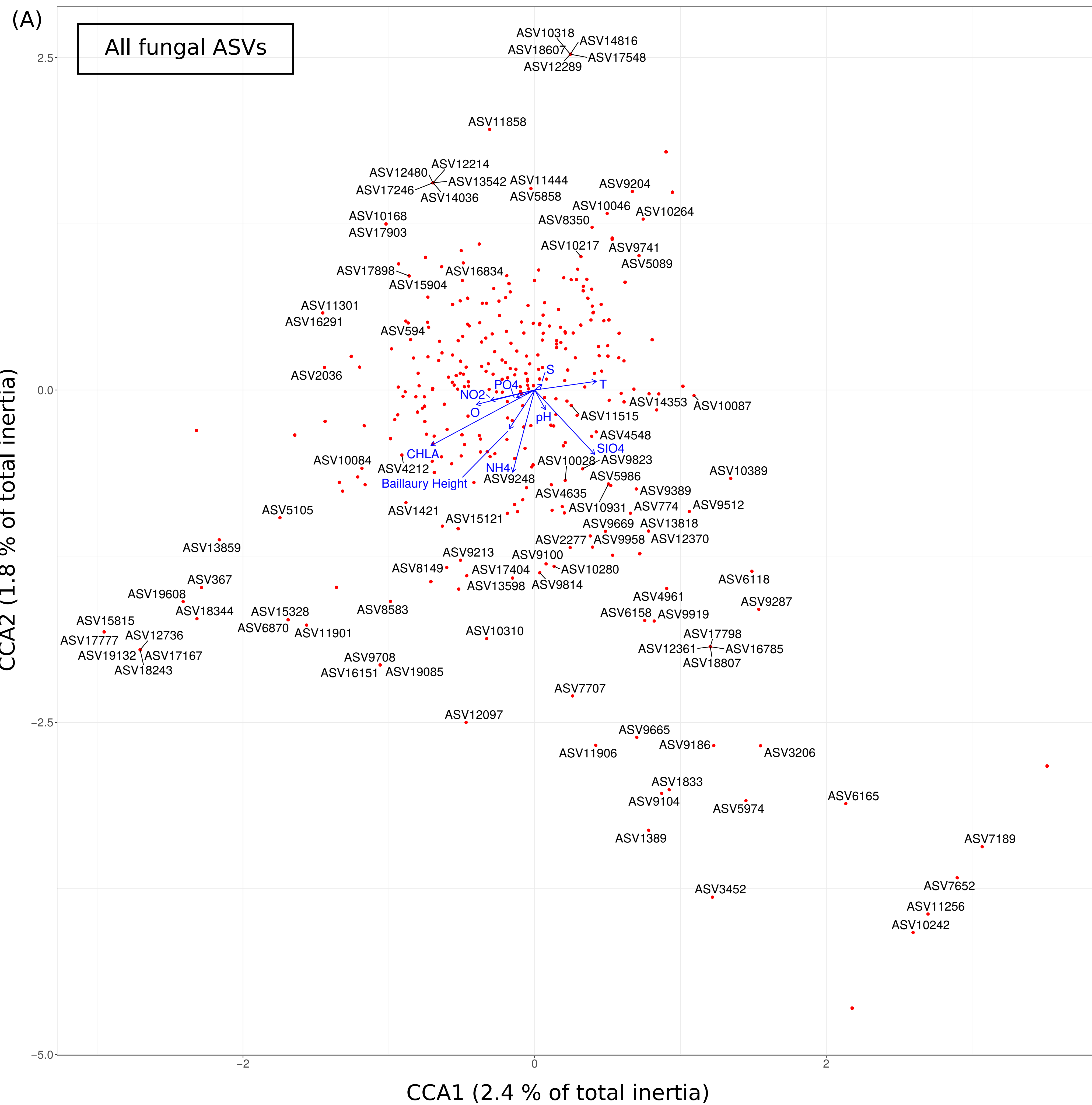

0.2 - 3  $\mu\text{m}$

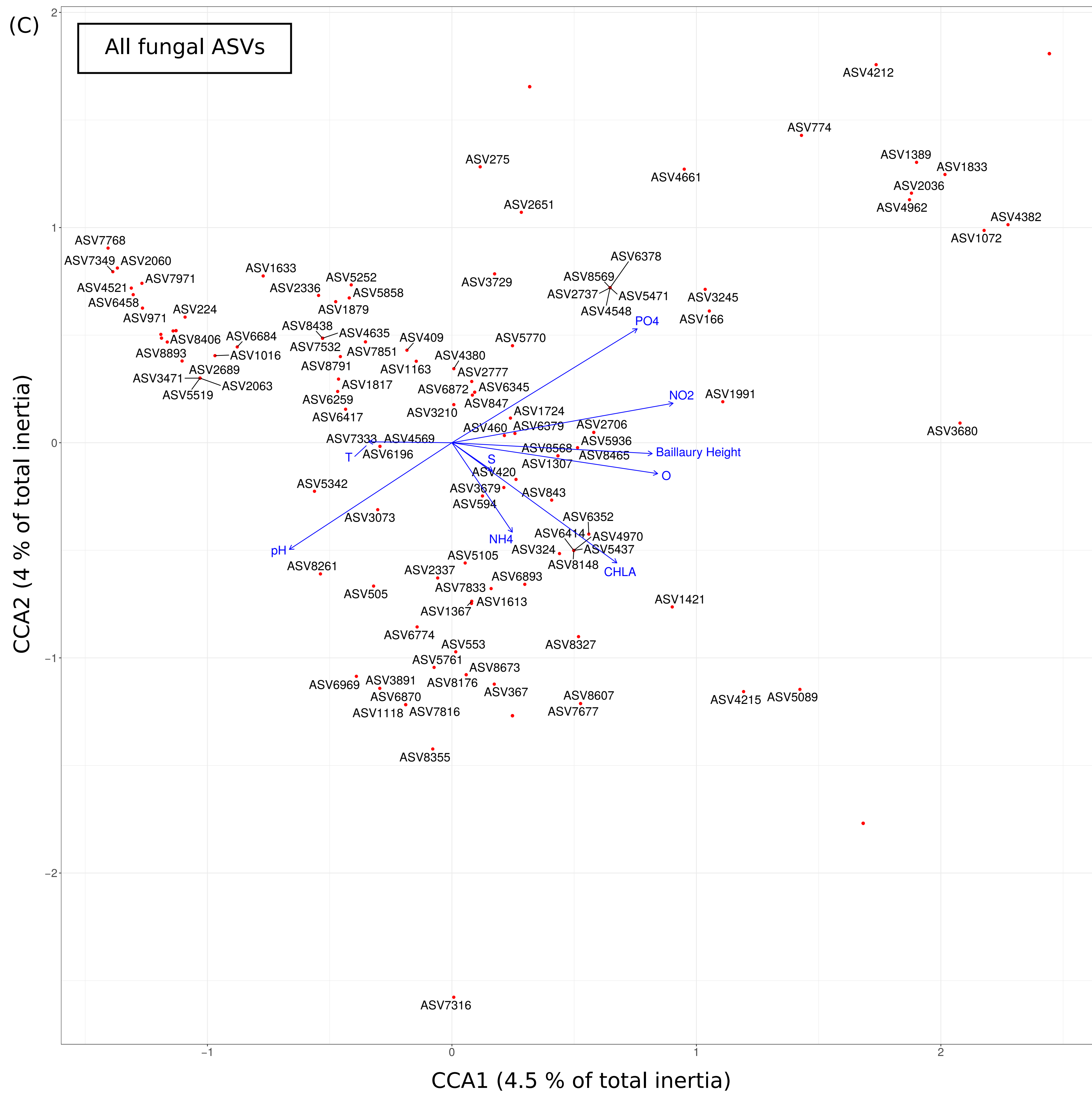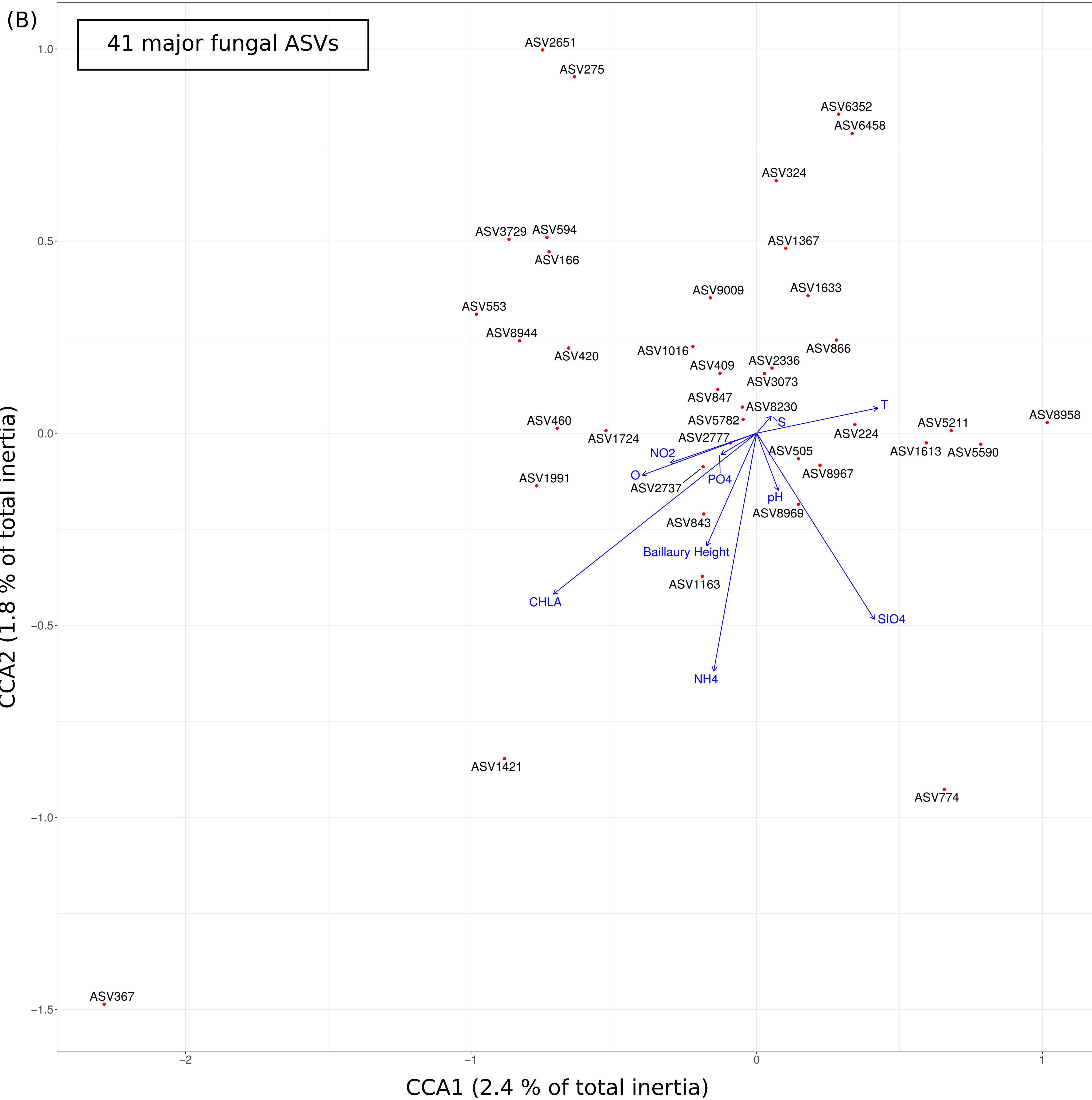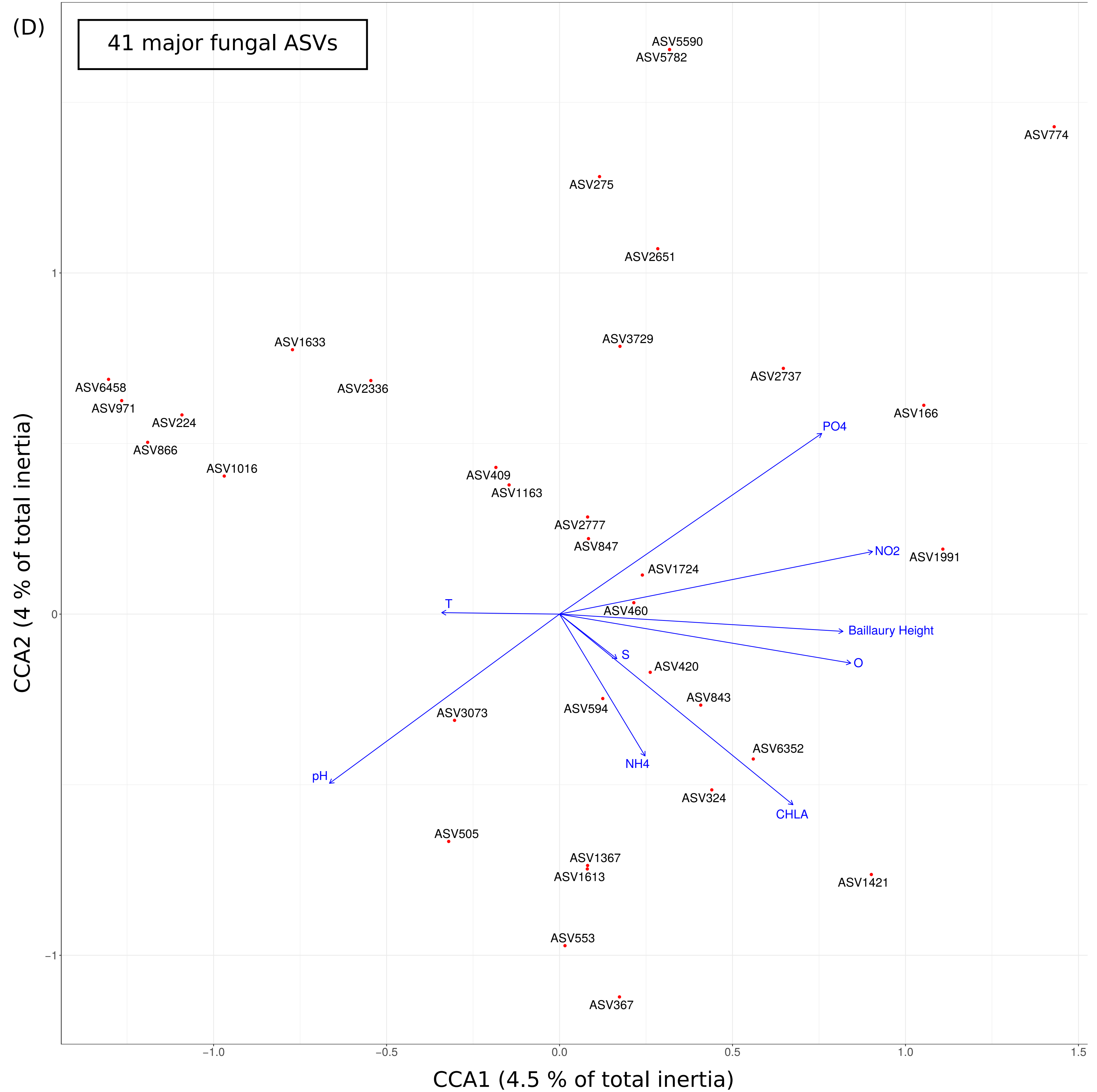

Supplement: Supplementary file 11 — Figure S11. Canonical Correlation Analysis (CCA) using the RAASVFungi of all fungal ASVs as response variables and environmental variables as explanatory variables using the samples from (A) the > 3 μm size fraction and (C) the 0.2–3 μm size fraction. (B) and (D) are respectively identical to (A) and (C), except that only the 41 major fungal ASVs are displayed for clarity. Only samples with at least 50 fungal reads were considered in calculations. Blue arrows denote the explanatory variables and red dots the response variables. The projection uses scaling 2: the angle between any couple of variables (regardless of being response or explanatory) reflect their correlation. T: Temperature. S: Salinity. O: Dissolved oxygen. CHLA: Chlorophyll a concentration. Baillaury height: height of La Baillaury River ~3.2 km south‐west of SOLA. [file EMI4-17-e70154-s007.pdf]

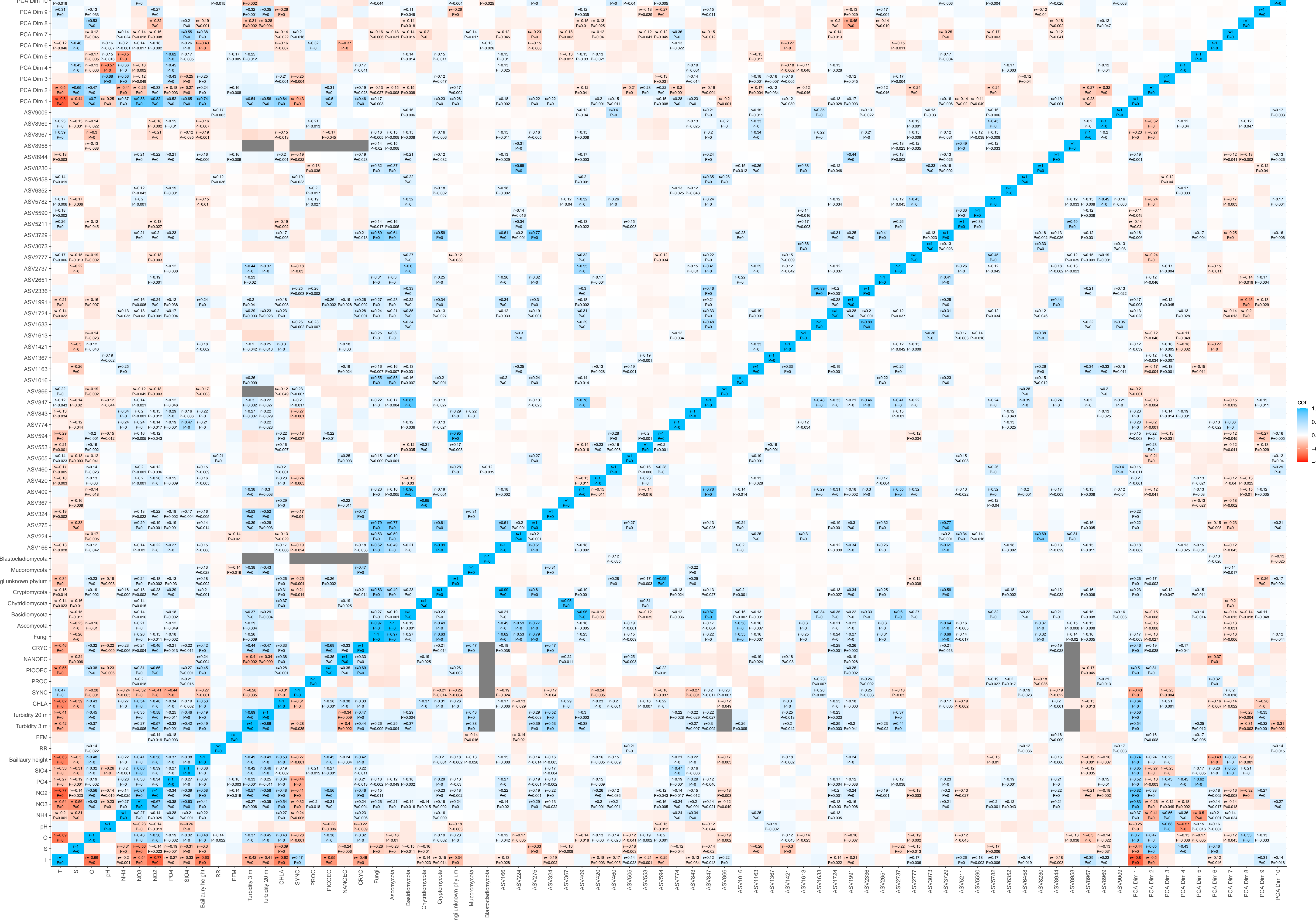

Supplement: Supplementary file 12 — Figure S12. Correlation (Pearson’s r) of all environmental variables, the biological variables, the first 10 dimensions of the PCA computed in Figure 2 and the relative abundance of Fungi, of fungal phyla, and of the 41 fungal ASVs that gather more than 90% of the cumulative relative abundance of Fungi. The colour denotes the correlation coefficient of the two variables. Grey tiles indicate the inability to calculate a correlation coefficient for these pairs of variables. The text in tiles indicates the correlation coefficient (r) and the associated p value (P) if the p value is significant (≤ 0.05). T: Temperature. S: Salinity. O: Dissolved oxygen. NH4: NH4 + concentration. NO3: NO3 − concentration. NO2: NO2 − concentration. PO4: PO4 3− concentration. SIO4: (SiO4)4− concentration. Baillaury height: the height of La Baillaury River ~3.2 km south‐west of SOLA. RR: Daily rainfall at Cape Béar. FFM: Average daily wind speed at 10 m at Cape Béar. Turbidity 3 m: Turbidity at 3 m below surface. Turbidity 20 m: Turbidity at 20 m below surface (~6 m above seafloor). CHLA: Chlorophyll a concentration. PROC, SYNC, PICOEC, NANOEC and CRYC indicate cell counts for respectively Prochlorococcus sp., Synechococcus sp., Pico‐eukaryotes, Nano‐eukaryotes and Cryptophyceae. Fungal group names (e.g., ‘Fungi’, ‘Ascomycota’, ‘ASV224’) refer to the relative abundance of each of these groups amongst eukaryotes. [file EMI4-17-e70154-s010.pdf]
